# Integrated Bioluminescent Immunoassays for High-Throughput Sampling and Continuous Monitoring of Cytokines

**DOI:** 10.1021/acs.analchem.3c00745

**Published:** 2023-05-30

**Authors:** Eva A. van Aalen, Bas J. H. M. Rosier, Tom Jansen, Simone F. A. Wouters, Robin T. Vermathen, Harmen J. van der Veer, José Yeste Lozano, Sheeza Mughal, Juan M. Fernández-Costa, Javier Ramón-Azcón, Jaap M. J. den Toonder, Maarten Merkx

**Affiliations:** † Laboratory of Chemical Biology, Department of Biomedical Engineering, Eindhoven University of Technology, P.O Box 513, 5600 MB Eindhoven, The Netherlands; ‡ Institute for Complex Molecular Systems, Eindhoven University of Technology, P.O Box 513, 5600 MB Eindhoven, The Netherlands; § Institute for Bioengineering of Catalonia (IBEC), The Barcelona Institute of Science and Technology (BIST), C/Baldiri Reixac 10-12, Barcelona E08028, Spain; ∥ Institució Catalana de Recerca i Estudis Avançats (ICREA), Passeig de Lluís Companys, 23,O Barcelona E08010, Spain; ⊥ Microsystems, Department of Mechanical Engineering, Eindhoven University of Technology, P.O Box 513, 5600 MB Eindhoven, The Netherlands

## Abstract

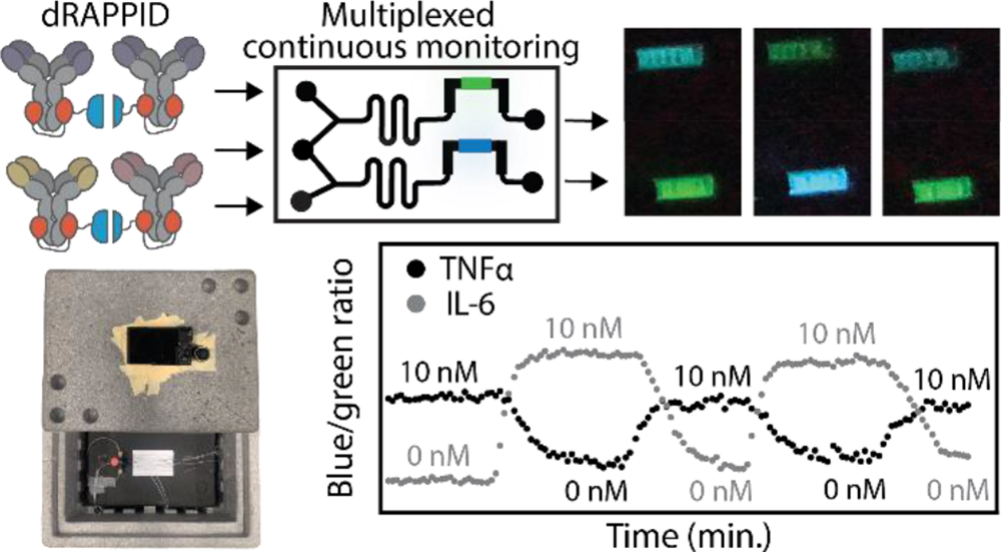

Immunoassays show great potential for the detection of low levels of cytokines, due to their high sensitivity and excellent specificity. There is a particular demand for biosensors that enable both high-throughput screening and continuous monitoring of clinically relevant cytokines such as interleukin-6 (IL-6) and tumor necrosis factor-α (TNFα). To this end, we here introduce a novel bioluminescent immunoassay based on the ratiometric plug-and-play immunodiagnostics (RAPPID) platform, with an improved intrinsic signal-to-background and an >80-fold increase in the luminescent signal. The new dRAPPID assay, comprising a dimeric protein G adapter connected via a semiflexible linker, was applied to detect the secretion of IL-6 by breast carcinoma cells upon TNFα stimulation and the production of low concentrations of IL-6 (∼18 pM) in an endotoxin-stimulated human 3D muscle tissue model. Moreover, we integrated the dRAPPID assay in a newly developed microfluidic device for the simultaneous and continuous monitoring of changes in IL-6 and TNFα in the low-nanomolar range. The luminescence-based read-out and the homogeneous nature of the dRAPPID platform allowed for detection with a simple measurement setup, consisting of a digital camera and a light-sealed box. This permits the usage of the continuous dRAPPID monitoring chip at the point of need, without the requirement for complex or expensive detection techniques.

## Introduction

Cytokines are small proteins that are secreted by cells to regulate complex signaling events. Proinflammatory cytokines, such as interleukin-6 (IL-6) and tumor necrosis factor-(TNF)α, play an important role in inflammation^1^ and are involved in inflammation-related diseases, such as in Crohn’s disease,^2^ atherosclerosis,^3^ and several types of cancer.^4−8^ Elevated cytokine levels in patients with cancer are often related to tumor expansion and differentiation and engender poor prognosis.^9−12^ Furthermore, cytokine concentrations can change over time due to disease progression or treatment, for example, in acute inflammation or sepsis.^13,14^ The levels of cytokines such as TNFα, IL-6, and IL-1β are elevated in sepsis, and their concentration profile is correlated with disease severity.^15−17^ Continuous detection of cytokine concentrations could aid clinicians in health monitoring and treatment selection and adjustment (i.e., personalized medicine).^13,17,18^ Besides applications in diagnostics, continuous monitoring of cytokines could improve our understanding of in vitro models such as organ-on-chip models, by providing temporally resolved information about the presence and concentration of relevant cytokines.^19−21^ In recent years, organs-on-chips have shown to be effective model systems for fundamental research in the mechanism of organ physiology as well as in determining therapeutic effectiveness, drug screening, and studying disease progression.^22−24^

Monitoring cytokine secretion is challenging due to the relatively small size of cytokines and the low physiological concentrations (picomolar range).^25−27^ Currently, ELISA and related (sandwich) immunoassays that rely on highly specific antibody-based target binding are widely used for the detection of cytokines.^26,28^ Although these heterogeneous methods allow for sensitive detection of analytes, they require a time-consuming workflow with several washing and incubation steps and expert operators.^29,30^ Therefore, ELISA is less suitable for tracking the dynamics of cytokine release nor adapted for high-throughput screening of cytokine levels. Alternative technologies that allow multiplex detection of cytokines in solution such as Luminex still require expensive and sophisticated detection technology.^31,32^ So far, few immunoassays have been developed that allow continuous monitoring of biomarkers. Soh and co-workers recently reported a fluorescence-based sandwich immunoassay for continuous monitoring of glucose and insulin by using a microfluidic device and bead-based immunoassays.^33^ Alternatively, the Prins lab applied the principles of antibody-based antigen detection based on the detection of single-particle mobility (biosensing by particle mobility, BPM).^34^

Bioluminescence-based detection shows great promise for sensitive continuous monitoring in point-of-need settings. Unlike fluorescence, which requires external excitation and suffers from autofluorescence and scattering, bioluminescence can be performed directly in complex samples and does not require dedicated detection equipment. The recently established bioluminescent ratiometric plug-and-play immunodiagnostics (RAPPID) platform relies on analyte-induced complementation of split NanoLuc (NLuc) luciferase fragments, which are covalently coupled to analyte-specific antibodies through photoconjugation with a protein G adapter.^35−38^ The simple mix-and-measure workflow of RAPPID does not require sample preparation or washing steps, thereby making the sandwich immunoassay easy to handle and enabling in-solution measurements. Furthermore, the assay can be performed with low sample volumes and allows for fast analyte detection, due to the fast binding kinetics of antibodies.

**Figure 1:**
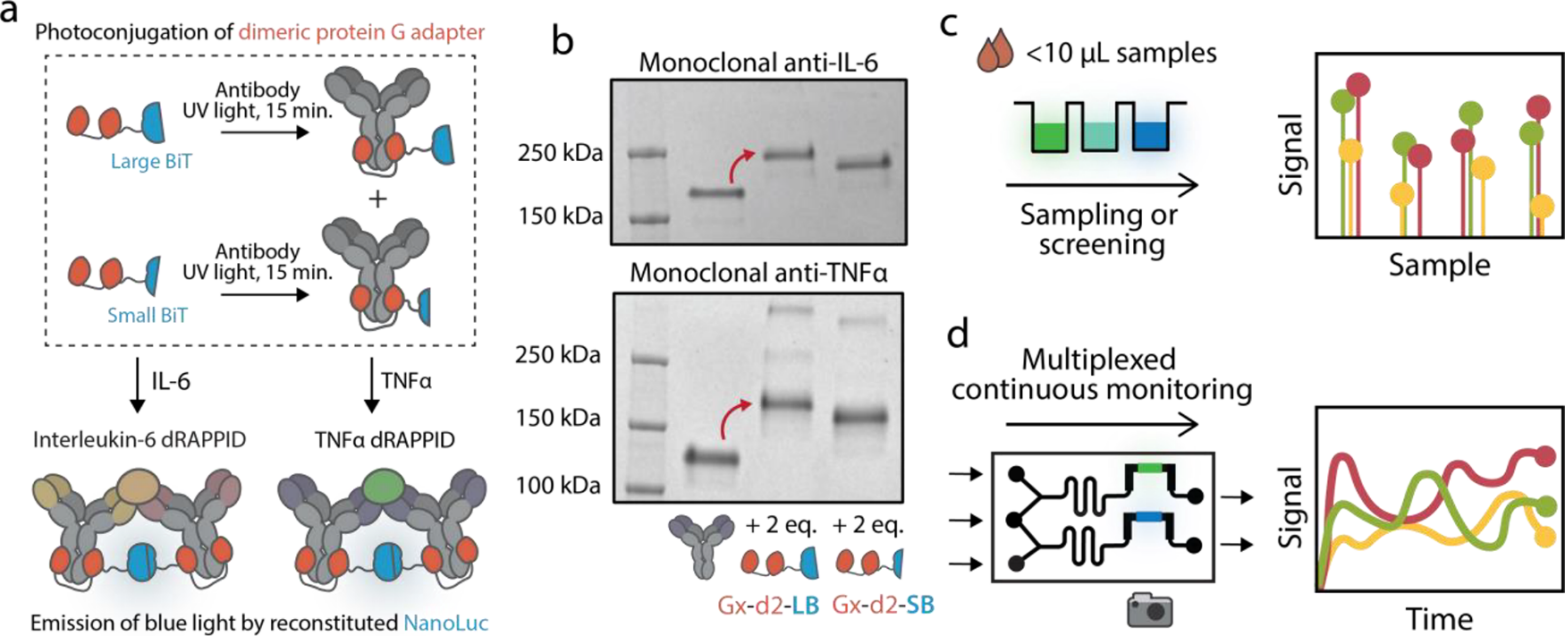
Development and implementation of the dimeric protein G RAPPID. (a) Schematic overview of the photoconjugation of the dimeric protein G adapters to anti-TNFα and anti-IL-6 antibodies. Reactions were performed in PBS (pH 7.4), with a 1:2 molar ratio of antibody to adapter, and were exposed to ultraviolet (UV) light (λ = 365 nm) for 15 min. (b) Nonreducing SDS-PAGE analysis of the photoconjugation described in (a), illustrating the efficient photoconjugation. The dimeric protein G RAPPID (dRAPPID) was applied to (c) detect the concentration of IL-6 in the culture medium from two different cell models and (d) continuously monitor the simultaneous presence of IL-6 and TNFα by using a herein-developed microfluidic chip.

Here, we adopted the RAPPID platform for highly sensitive detection of the cytokines TNFα and IL-6. To this end, we introduced a new dimeric protein G adapter that binds to both Fc domains on a single antibody, which increases photoconjugation efficiency and yields more homogeneous antibody-sensor conjugates (Figure 1a,b). Importantly, we show that the dimeric adapters reduce background luminescence which results in an increase in sensitivity. This dimeric RAPPID format (dRAPPID) was subsequently used to measure subnanomolar IL-6 levels both in traditional cell cultures of breast cell lines and in tissue-engineered 3D muscle models (Figure 1c). Furthermore, we designed and developed a dedicated microfluidic chip that allows continuous monitoring of analyte concentrations by enabling continuous mixing of sensor components of the dRAPPID assay with samples. We applied this continuous monitoring chip for the multiplexed continuous monitoring of TNFα and IL-6 (Figure 1d).

## Experimental Section

### Cloning

pET28 plasmids encoding Gx-d2-LB and Gx-d2-SB were obtained by performing overhang PCR and subsequent restriction using XhoI, KpnI, and SacI (Figures S2 and S3). Successful cloning was confirmed via Sanger Sequencing (BaseClear).

### Protein Expression

dRAPPID sensors were expressed as described in ref (35). Briefly, *Escherichia coli* BL21 (DE3) cells were transformed with pET28 plasmids encoding Gx-d2-LB or Gx-d2-SB together with a pEVOL vector (a kind gift from Peter Schultz, Addgene plasmid # 31190) encoding a tRNA/tRNA synthetase pair to incorporate the unnatural amino acid para-benzoyl-phenylalanine (pBpA, Bachem, 104504-45-2) at the amber stop codons.^39^ The cells were cultured at 20 °C overnight, subsequently harvested by centrifugation, and lysed using Bugbuster and benzonase (Novagen). Purification of the expressed Gx-d2-LB and Gx-d2-SB was done using Ni^2+^ affinity chromatography followed by Strep-Tactin purification. The purity of the two proteins was determined using SDS-PAGE analysis (Figure S4). The correct incorporation of two unnatural amino acids was confirmed by Q-ToF LC–MS (Waters MassLynx v4.1), using MagTran v1.03 for MS deconvolution. The observed mass for the Gx-d2-LB and Gx-d2-SB was 45862.81 Da (expected: 45863.04) and 29593.36 Da (expected: 29593.52), respectively.

### Photoconjugation

Gx-d2-SB and Gx-d2-LB were photoconjugated to infliximab (obtained via the Máxima Medisch Centrum pharmacy in Veldhoven, The Netherlands) and anti-IL-6 (10395-mhK23 and 10395-R508, ordered from SinoBiological). Photoconjugation was performed with 1 μM antibody and 2 μM Gx-d2-SB or Gx-d2-LB in PBS (pH 7.4) for 15 min, by using a Thorlabs M365LP1 (λ = 365 nm) UV-lamp coupled with a Thorlabs LEDD1B T-Cube LED Driver (continuous wave, current limit 1.2A, 80% intensity), as shown in Figure S5. After conjugation, the crosslinked sensor components were not further purified and stored at 4 °C until use.

### dRAPPID Assays

The dRAPPID assays were done in buffer (PBS (pH 7.4), 0.1% (w/v) BSA) or in Dulbecco’s modified Eagle’s medium (DMEM, from Gibco) with phenol red, supplemented with 4.5 g/L d-glucose, 0.58 g/L l-glutamine, 10% fetal bovine serum (FBS), 100 U/mL penicillin, and 100 μg/mL streptomycin (all from Life Technologies). An assay mixture of 1 nM antibody-LB and 10 nM antibody-SB was incubated with a target analyte for 60 min prior to the addition of 1000-fold diluted NLuc substrate (Promega, N1110). For ratiometric detection, 50 or 17 pM of calibrator luciferase was added to the sensor mixture for IL-6 or TNFα detection, respectively. The assays were executed in nontreated white Thermo Scientific 384-well plates (Cat. no 262360) in a total volume of 20 μL. The luminescent signals were recorded between 398 and 653 nm on a Tecan Spark 10 M plate reader, with a 25 nm bandwidth, at room temperature. The blue-to-green ratios were calculated by dividing the blue light emission at 458 by the green light emission at 518 nm. Detection with a digital camera (Sony DSC-Rx100 III) was done in a gray Styrofoam box. The LOD was calculated using eq 1, in which SD is the standard error of the *y*-intercept, by linear regression of the luminescent signal related to a selection of low cytokine concentrations.
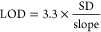
1

### Off-Line dRAPPID Assay of Secreted IL-6

MDA MB 231 and MCF-7 cells were cultured in DMEM (with phenol red, supplemented with 4.5 g/L d-glucose, 0.58 g/L l-glutamine, 10% fetal bovine serum (FBS), 100 U/mL penicillin, and 100 μg/mL streptomycin) in Falcon corning T25 culture flasks (Nunc EasYFlask Cell Culture Flasks) at 37 °C with 5% CO_2_ until a confluency of 80% was reached. Subsequently, 200,000 cells were seeded in a CELLSTAR 12-well cell culture plate (Greiner, cat.-no. 665180) and grown overnight. The next day, the cell culture medium was removed and fresh DMEM, with 0.5 nM TNFα or without TNFα, was added to the cells. After 0, 6, 12 and 24 h, 10 μL culture medium samples were removed from the cells and IL-6 dRAPPID (1 nM antibody-LB and 10 nM antibody-SB) was added to these culture medium samples. After an incubation step of 60 min, the substrate of NLuc was added (1000-fold diluted) and the luminescent signal was measured on a Tecan Spark 10 M plate reader. Culturing of the 3D muscle tissues was performed according to ref (49). The muscle tissues were stimulated with 10 μg/mL lipopolysaccharide (LPS) or left untreated. Culture medium samples (10 μL) were removed from the muscle tissues after 2, 4, and 24 h of LPS stimulation and mixed with 0.1 nM anti-IL-6-LB and 1 nM anti-IL-6-SB. After 1 h of incubation, 1000-fold diluted NLuc substrate was added, and the luminescent signal was measured with a plate reader (Infinite 200Pro). To test for significant differences in IL-6 secretion between TNFα stimulated and nonstimulated cells at the same timepoint, and IL-6 secretion between different timepoints in LPS-stimulated and nonstimulated tissues, a paired t-test was used. Statistical analysis was performed in MATLAB (Mathworks, USA).

### Development of Microfluidic Devices

The photolithography mask was designed in AutoCAD software (Figure S6) and then printed via CAD/Art Services, Inc. The SU-8 master wafer was fabricated by patterning ∼100 μm-thick SU-82075 photoresist (MicroChem) on a silicon wafer. UV exposure was done with an exposure energy of 230 mJ/cm^2^ and an irradiation of 20 mW/cm^2^. The development of the wafer was performed for 30 min in Mr. Developer 600 (Microresist, Germany). The final polydimethylsiloxane (PDMS) chip was fabricated by soft lithography. Accordingly, SYLGARD 184 base and curing agent (Dow, USA) were mixed in a 10:1 ratio (w/w) and placed in a vacuum desiccator to remove air bubbles. The mixed and degassed mixture was poured over the SU-8 master wafer to form a ∼5 mm-thick PDMS layer. The PDMS was subsequently cured at 65 °C for 6 h. After this curing step, the PDMS chip was removed from the SU-8 wafer and the inlet and outlet holes were punched with a 1.2 mm puncher (Uni-Core). To bond the PDMS to a glass slide (24 × 50 mm), plasma treatment (in a Quorum K1050X asher) was used. Plasma treatment of the glass slide and PDMS chip was done with oxygen plasma for 30 s with an RF power of 20 W. Immediately after treatment, the glass and PDMS were brought into contact and placed at 65 °C for 1 h. Before performing the continuous monitoring experiments, the detection chambers, 6 mm of polyethylene tubing (from scientific Commodities Cat. Nr. BB31695-PE/5, with an inner diameter of 0.034 inch) and two metal adapters, were added into the punched holes.

### Continuous Measurements in Microfluidic Chips

Food coloring experiments were performed in buffer (PBS (pH 7.4), 0.1% (w/v) BSA) with red food coloring (TRS Foods, UK, 25 mg/mL). PHD ULTRA CP Syringe Pumps (Harvard Apparatus) and 1 mL syringes (Henke-Ject) were used to inject buffer with food coloring (analyte inlet) and buffer without food coloring (sensor inlets and NLuc substrate inlet) into the microfluidic chip, with flowrates of 1, 2, and 4 μL/min for the sensor inlet, analyte sample inlet, and NLuc substrate inlet, respectively. The microfluidic chip was placed in a light-proof black Styrofoam box and polyethylene tubing (from Scientific Commodities) was used to connect the chip to the syringes in the pumps. The sensor, analyte, and substrate were injected for 30 min at these flow rates before measurements started, to ensure steady flow in the chip. Pictures of detection chambers were taken with a Sony DSC-Rx100 III digital camera, with an integration time of 30 s and an ISO value of 6400. Continuous monitoring experiments with dRAPPID were executed as described above but with 1 nM of antibody-LB, 10 nM of antibody-SB, 250-fold diluted NLuc substrate, and 60 or 27 pM of calibrator luciferase for IL-6 dRAPPID or TNFα RAPPID, respectively. Switching between 0 nM analyte and 10 nM analyte (both in PBS (pH 7.4), 0.1% (w/v) BSA) was done using a IDEX V-101D manual flow switching valve.

## Results and Discussion

### Development of Dimeric RAPPID for IL-6 and TNFα Detection

The classic RAPPID platform comprises one photo-cross-linkable protein G adapter domain (Gx) connected via a semiflexible linker to either large BiT (LB) or small BiT (SB), the two fragments of split NLuc.^36^ Subsequent crosslinking to antibodies typically generates a mixture of non-, once-, and twice-conjugated antibodies, resulting in background luminescence by undesired NLuc complementation due to binding of a free Gx-LB or Gx-SB to an unoccupied heavy chain (Figure S7). To further reduce the background signal and enable the detection of low concentrations of cytokines, we here established a new class of dimeric RAPPID (dRAPPID) sensors. The dRAPPID sensor proteins consist of two photo-cross-linkable protein G adapters, connected via a semiflexible linker that was designed to optimally span the distance between the two heavy chains of one antibody, fused to an LB or an SB (*K*_D_ = 2.5 μM) domain.^36^ The Gx-d2-SB and Gx-d2-LB proteins were expressed in *Escherichia coli* and purified using Ni^2+^ affinity chromatography followed by Strep-Tactin purification (Figure S4). The sensor components and the monoclonal anti-IL-6 or anti-TNFα antibodies were mixed in a 1:2 molar ratio, ensuring the binding of the two protein G adapters of one sensor component to the Fc-domain of an antibody in a 1:1 stoichiometry. Subsequent short UV light irradiation (15 min, λ = 365 nm) of the mixture results in the covalent crosslinking of antibody to the sensor (Figures 1a and S5). SDS-PAGE analysis showed an improved photoconjugation efficiency and a more homogeneous reaction mixture compared to classic RAPPID sensors with only one protein G adapter (Figures 1b and S7a).^33,35^ The two minor bands corresponding to higher molecular weight species in Figure 1b can be attributed to two sensor components conjugated to one antibody and one sensor protein photo-crosslinked to two antibodies. Furthermore, in this new dRAPPID platform, both heavy chains of the IgG antibody are occupied by a protein G adapter, preventing the binding of a protein G-dimer with the complementary fragment of split NLuc and thus resulting in less background luminescent activity (compare Figure S7b with Figure S7c). This low background signal is particularly important for the detection of analytes such as cytokines, which are generally present in low concentrations.

**Figure 2:**
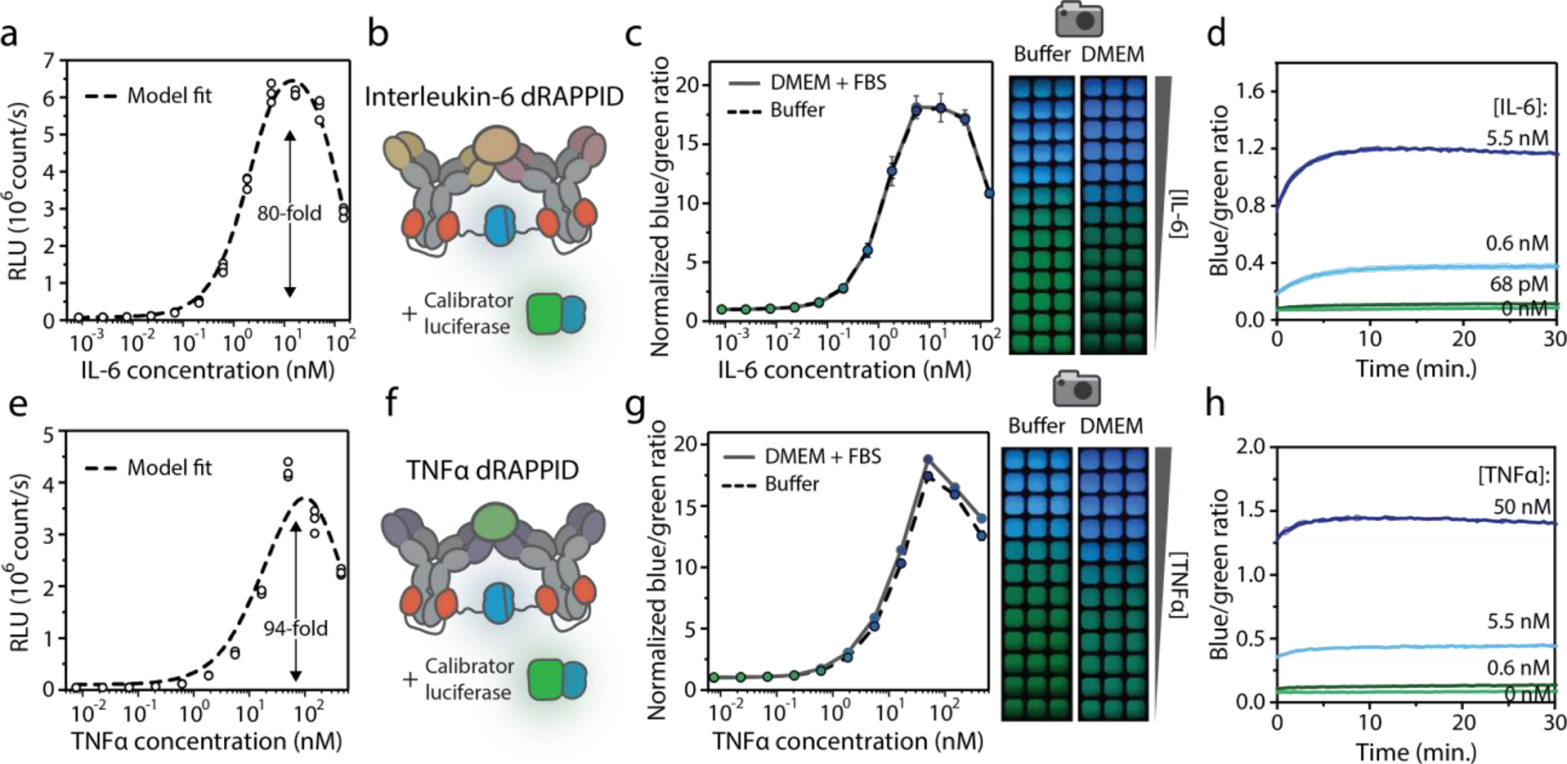
Performance of the protein G-dimer RAPPID assays. (a) Intensiometric detection of IL-6 in buffer (PBS (pH 7.4), 0.1% (w/v) BSA). RLU, relative luminescence units. (b) Schematic overview of the ratiometric IL-6 dRAPPID assay. (c) Ratiometric detection of IL-6 in buffer (PBS (pH 7.4), 0.1% (w/v) BSA) or in the cell culture medium (DMEM with 10% FBS) with 50 pM concentration of the calibrator luciferase. The same samples were used for detection with the plate reader (left) and with a digital camera (right). (d) Ratiometric sensor output over time at various concentrations of IL-6. All components (sensors, IL-6, substrate, and calibrator luciferase) were added at *t* = 0. (e) Intensiometric TNFα dRAPPID in buffer (PBS (pH 7.4), 0.1% (w/v) BSA). RLU, relative luminescence units. (f) Schematic overview of the ratiometric TNFα dRAPPID assay. (g) Ratiometric detection of TNFα in buffer and DMEM (+10% FBS) with 17 pM calibrator luciferase. The measurements in the plate reader (left) and the pictures with the digital camera (right) were done with the same samples. (h) Ratiometric response over time after one-step incubation of all TNFα dRAPPID components. The reaction mixtures in (a), (c), (e), and (g) consisted of 1 nM Ab-LB and 10 nM Ab-SB and were incubated for 60 min at room temperature prior to the addition of 1000× diluted NLuc substrate. Data points in (a) and (e) represent technical replicates, with *n* = 3 independent preparations of the target analyte. Experimental data were fitted to a thermodynamic model (dotted lines, Supporting Information). Values in (c) and (g) depict mean ± s.d. of technical replicates, with *n* = 3 independent preparations of the target, and the lines represent the mean. Lines in (d) and (h) represent mean ± s.d. of technical replicates, with *n* = 3 independent preparations of the analyte.

After confirming the efficient photoconjugation of the sensor components to the IL-6 antibodies (using a commercially available ELISA-pair binding distinct epitopes), we tested the performance of the new IL-6 dRAPPID sensor in PBS buffer. Increasing concentrations of IL-6 were added to 1 nM of anti-IL-6 Gx-d2-LB (Ab-LB) and 10 nM of anti-IL-6 Gx-d2-SB (Ab-SB) and incubated for 60 min at room temperature. Subsequently, the furimazine substrate of NLuc was added and the luminescent signal was measured with a plate reader. Binding of the dRAPPID sensor components to the IL-6 target resulted in NLuc complementation and an IL-6-dependent increase in blue light emission. Figure 2a shows that the IL-6 dRAPPID exhibited an 80-fold increase in the luminescent signal and the detectable IL-6 concentration regime spanned three orders of magnitude. At IL-6 concentrations exceeding ∼10 nM, the luminescent signal decreased again due to the hook effect.^35^ Fitting a thermodynamic model of the system generated a bell-shaped curve that closely resembled the data in Figure 2a and yielded antibody affinities of ∼2.3 and ∼120 nM for 10395-mhK23(-SB) and 10395-R508(-LB) (Figure S1a).^40^ Next, we translated this intensiometric IL-6 dRAPPID assay into a ratiometric version by adding the green light-emitting calibrator luciferase, a genetic fusion of NLuc to mNeonGreen (Figure 2b).^35,41^ The ratio of blue light, emitted by the complemented Nluc of the sensor components, and green light of the calibrator luciferase gives a stable ratiometric signal that is insensitive to changes in environmental factors such as pH and temperature and does not suffer from the signal decrease over time as a result of substrate depletion. Figure 2c displays the performance of the ratiometric IL-6 dRAPPID under standard buffer conditions (PBS (pH 7.4), 0.1% (w/v) BSA) and in the cell culture medium (DMEM with 10% FBS). dRAPPID performed in buffer and DMEM yielded similar bell-shaped dose–response curves and the IL-6-dependent blue-to-green ratios were detectable both with a plate reader and with a simple digital camera. The kinetics of the complex formation between dRAPPID components and IL-6 was fast, reaching a stable blue-to-green ratio after ∼10 min (Figure 2d).

Next, we analyzed the performance of the new TNFα dRAPPID assay, consisting of the same anti-TNFα antibody (infliximab) conjugated to either Gx-d2-LB or Gx-d2-SB. The sensor components were incubated for 60 min (1 nM Ab-LB and 10 nM Ab-SB), to ensure complex formation, with increasing concentrations of TNFα. Figure 2e shows that this intensiometric TNFα dRAPPID assay exhibited a dose–response curve with a maximal 94-fold increase in luminescent signal. Fitting of a thermodynamic model to the intensiometric titration data yielded an antibody affinity of ∼155 nM for TNFα (Figure S1b). The ratiometric TNFα assay displayed a ∼19-fold increase in the blue-to-green ratio, both in buffer and culture medium, upon increasing concentrations of TNFα (Figure 2f,g). The ratiometric signal reached a stable signal within ∼5 min, demonstrating fast complex formation between TNFα and the sensor components (Figure 2h). Furthermore, by decreasing the concentration of the dRAPPID sensor components to 0.1 nM of Ab-LB and 1 nM of Ab-SB, the limit-of-detection could be lowered to 4.5 and 18 pM for IL-6 and TNFα, respectively (Figure S8). Taken together, these results show that the new dimeric protein G adapter RAPPID platform allows for fast and sensitive detection of cytokines, due to low background luminescence, and performs excellent both in buffer and culture media.

### Sampling of Cell-Secreted Interleukin-6

**Figure 3:**
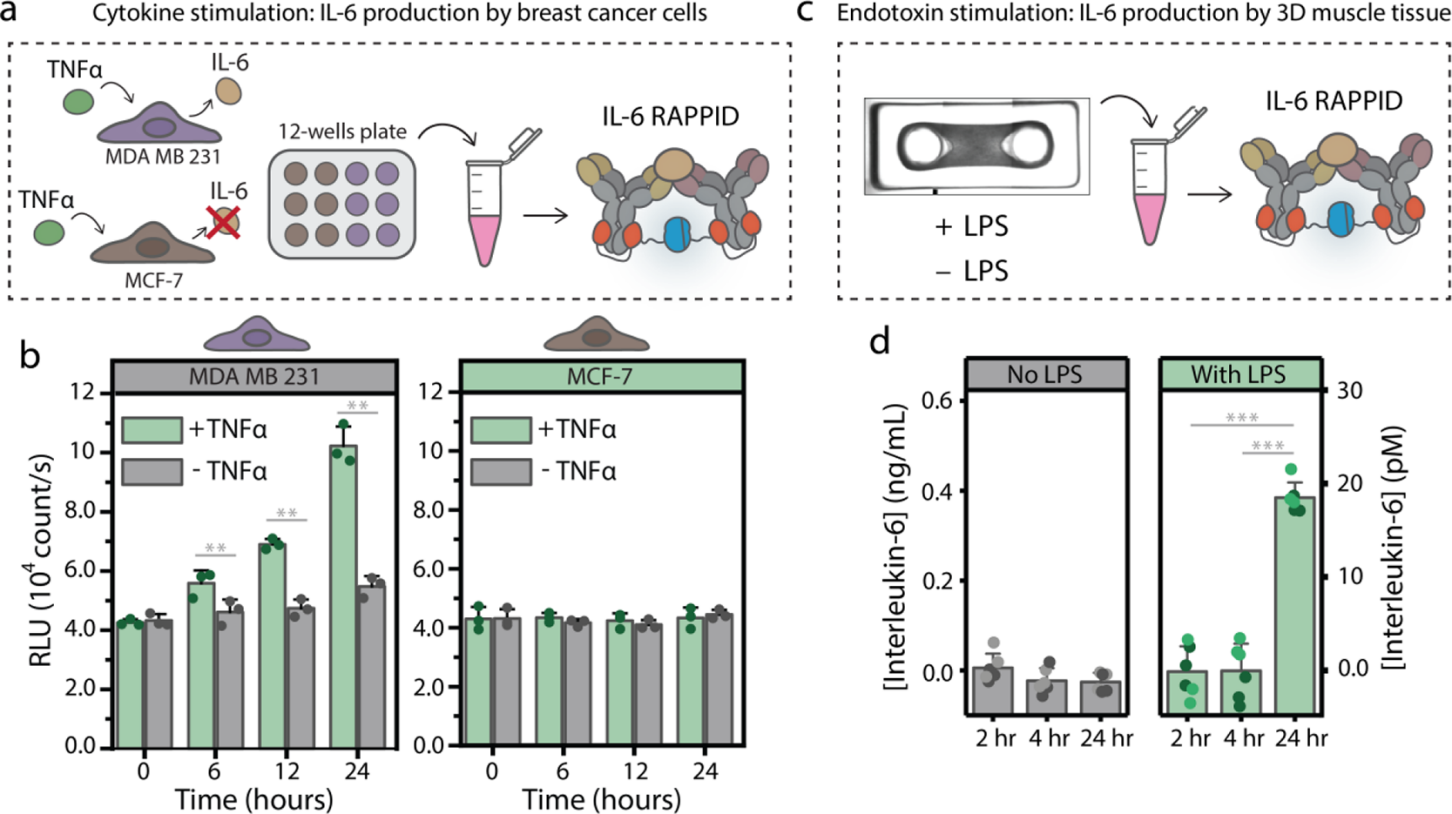
Off-line measurements of IL-6 in the cell culture medium. (a) Schematic representation of IL-6 detection in the cell culture medium of cytokine-stimulated breast cancer cells. MDA MB 231 and MCF-7 cells were cultured in a 12-well plate and were either stimulated with 0.5 nM TNFα or left untreated. Culture medium samples were taken after 0, 6, 12, and 24 h of TNFα stimulation and measured with IL-6 dRAPPID (1 nM Ab-LB and 10 nM Ab-SB) in a plate reader after 60 min of incubation. (b) IL-6 detection in the cell culture medium of MDA MB 231 and MCF-7 cells. Individual data points (*n* = 3) are shown, and bars represent mean values with s.d. RLU, relative luminescence units. (c) Schematic representation of IL-6 detection in the culture medium of human 3D muscle tissue. Muscle cells were cultured in a hydrogel in a PDMS mold, and cells were stimulated with 10 μg/mL lipopolysaccharide (LPS) or left untreated. IL-6 dRAPPID (0.1 nM Ab-LB and 1 nM Ab-SB) was added to the cell culture medium, and after 60 min, of incubation the luminescent signal was measured with a plate reader. (d) IL-6 detection with dRAPPID of the 3D human muscle tissue. *n* = 2 biological replicates, *n* = 3 technical replicates and bars represent mean ± s.d. Asterisks represent a significant difference (** = *p* ≤ 0.01, *** = *p* ≤ 0.001).

The low background signal of the new class of RAPPID variants, with the ensuing low limit-of-detection, makes dRAPPID well suited to measure the low concentrations of cytokines secreted by cells. Furthermore, the excellent performance in the culture medium, the minimal sample handling, and the mix-and-measure workflow makes the dRAPPID assay a promising tool for high-throughput screening. To demonstrate this, we measured IL-6 secretion levels after stimulation with a cytokine or an endotoxin, for two different cell models. First, we used dRAPPID to detect IL-6 secretion of breast carcinoma cells after cytokine stimulation. MDA MB 231 cells are highly invasive cancer cells and are known to have an upregulated and cytokine-responsive IL-6 expression compared to other breast cancer cell lines such as MCF-7 that do not respond to this type of stimulation.^42−44^ MDA MB 231 and MCF-7 cells were cultured in a 12-well plate, stimulated with TNFα or left untreated, and samples of the cell culture medium were taken at different time points (*t* = 0, 6, 12, and 24 h). The levels of secreted IL-6 in these samples were subsequently determined by adding IL-6 dRAPPID and measuring the corresponding luminescent signal (Figure 3a). Figure 3b shows that stimulation with TNFα resulted in the accumulation of IL-6 in the cell culture medium of the MDA MB 231 cells, demonstrated by the increase in luminescent signal over time. Untreated MDA MB 231 cells did not display this increase in luminescent signal, suggesting that stimulation is needed for elevated IL-6 secretion of these cancer cells. As expected, the MCF-7 cells did not secrete IL-6 upon TNFα stimulation, as the luminescent signal stayed constant and did not increase over time (Figure 3b).

A 3D tissue construct more accurately represents the cells in an in vivo environment and is hence a better model to study topics like disease physiology and drug discovery than a more traditional 2D cell culture.^45,46^ To investigate the compatibility of dRAPPID with advanced cell culturing systems, we stimulated a human 3D muscle tissue model with lipopolysaccharide (LPS) and measured the corresponding IL-6 secretion of the muscle cells with dRAPPID. LPS is an endotoxin that stimulates the expression of proinflammatory cytokines in many different types of tissues and cells, including skeletal muscle cells.^47,48^ We fabricated the 3D muscle models as described previously and subsequently stimulated them with LPS.^49^ Culture medium samples (10 μL) were removed after 2, 4, and 24 h of LPS stimulation (Figure 3c). After analyzing these samples with IL-6 dRAPPID, we found that after 24 h of LPS stimulation, the muscle tissue secreted ∼18 pM (corresponds to ∼0.4 ng/mL) of IL-6 (Figure 3d). Muscle tissue without LPS stimulation did not show an increase in secreted IL-6 levels over time. The low-picomolar sensitivity of the dRAPPID platform would also allow the detection of IL-6 levels secreted by other in vitro cell models such as monocytes, astrocytes, adipose tissue, and muscle tissues, where accumulated concentrations can reach low-picomolar to mid-picomolar levels.^21,50−52^ Furthermore, the dRAPPID assay allows the quantification of analytes in volumes as low as 10 μL, making high-throughput screening possible in situations where high temporal resolution is necessary or when screening of many conditions in parallel is desired.

### Development of a Microfluidic Chip for Continuous Monitoring

**Figure 4:**
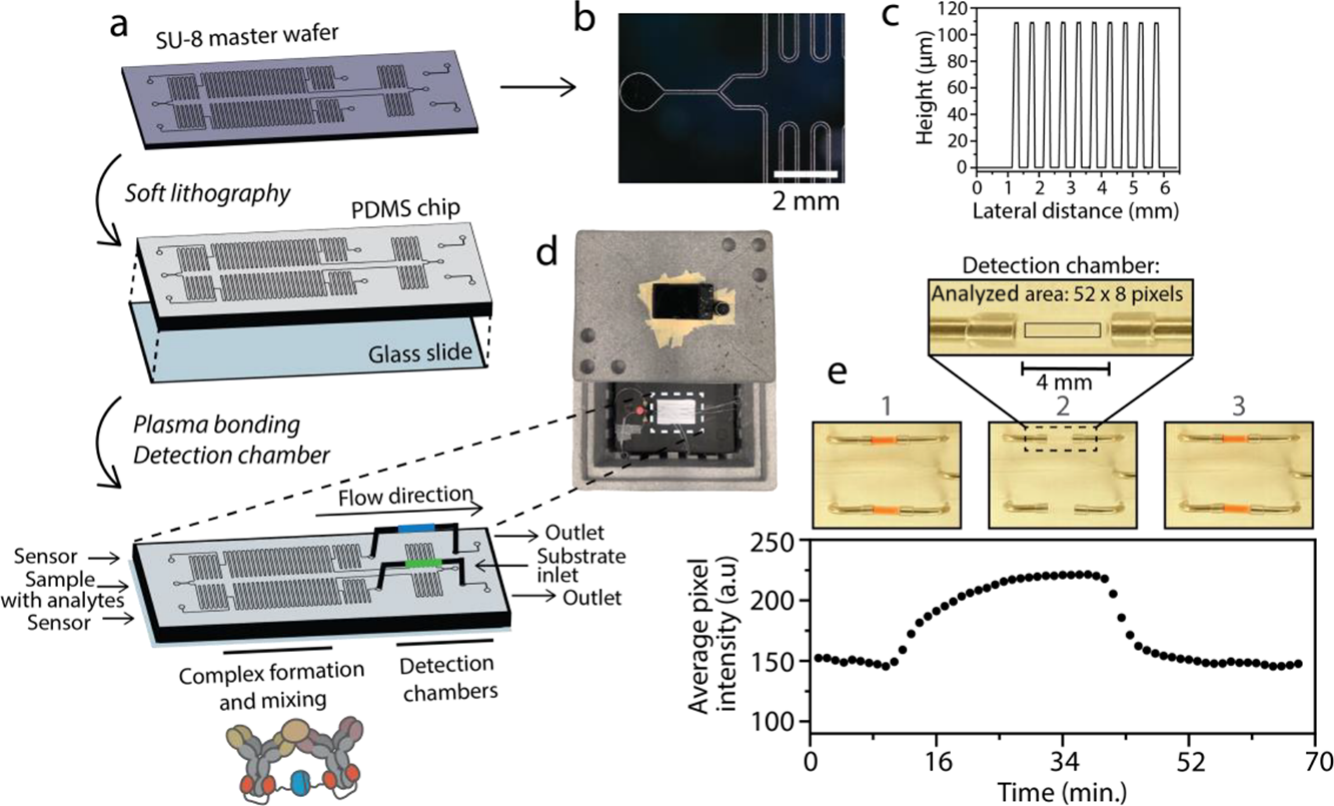
Fabrication and testing of the continuous monitoring microfluidic chip. (a) Fabrication of the microfluidic chip using photolithography and soft lithography. The microfluidic chip has two sensor inlets, one sample inlet, one NLuc substrate inlet, and two outlets. Complex formation and mixing of the analytes and the sensors take place in the long serpentine channel, and detection of the luminescent signal occurs in the detection chambers with a digital camera. (b) Digital microscope image of the SU-8 master wafer. (c) Contact profilometer analysis of the microchannels of the SU-8 master wafer. (d) Measurement setup, consisting of a light-tight box in which the chip is positioned and that has a hole for placing a conventional digital camera (Sony DSC-RX100 III). (e) Performance of the continuous monitoring chip. Switching between different analyte inputs, buffer (PBS (pH 7.4), 0.1% (w/v) BSA) without and buffer with red food coloring (25 mg/mL), is done with a V-101D flow switching valve. Switching occurred at *t* = 1 min and *t* = 25 min. Flow rates of 1, 2, and 4 μL/min for sensor inlets, analyte sample inlets, and NLuc substrate inlets, respectively, were used. Data represent the average pixel intensity measured in the analyzed area (52 × 8 pixels, shown in the inset) of the detection chamber.

The continuous monitoring of cytokines shows potential in both healthcare and research applications. Hence, we developed a microfluidic device for continuous sensing and integrated dRAPPID for IL-6 and TNFα detection. Accordingly, we used a standard microfluidic device fabrication protocol with an SU-8 master wafer, soft lithography, and sealing with a glass slide to develop the polydimethylsiloxane (PDMS) microfluidic chip shown in Figure 4a. Microscope inspection of the SU-8 master showed the correct fabrication of the microchannels (Figure 4b) and measurements with a contact profilometer confirmed that the features had the desired height of ∼100 μm (Figure 4c). Because the signal strength of the bioluminescent output is directly related to the *z*-height of the detection chamber, we decided to implement external detection chambers consisting of polyethylene tubing and two metal adapters to obtain the fully assembled microfluidic chip (Figure S9). The PDMS chip contained four different inlets, two intended for dRAPPID sensors, enabling multiplexed detection of two different biomolecules, one inlet for the sample containing the analytes of interest and one for the substrate of NLuc (Figure 4a). After the sample has entered the microfluidic chip, the fluid splits and mixes with the different dRAPPID sensors in the long serpentine channels, resulting in the formation of a complex between the target and sensor. Before the sensor-analyte mixture enters the detection chamber, it is mixed with the substrate of NLuc and the luminescent signal of the reconstituted NLuc, indicating the presence of the target analyte, can subsequently be observed in the detection chambers. The detection of this luminescent signal is done by using a simple light-tight box with a hole for a digital camera, enabling continuous monitoring of biomolecules without expensive and sophisticated equipment (Figure 4d). New dRAPPID sensors and samples are continuously injected into the chip, refreshing the detection chamber with newly formed complexes and thereby enabling the continuous detection of changes in analyte concentration.

Using optimized flowrates (Figure S10), which enabled ∼3 min of complex formation between sensor and analyte in the serpentine channels, we next determined the performance and the temporal resolution of the microfluidic chip by injecting buffer samples with or without red food coloring (Figure 4e). We started by injecting buffer with red food coloring in the analyte inlet (1 in Figure 4e), switched to buffer without food coloring, resulting in the disappearance of the red color (2 in Figure 4e), and finally switched back to buffer with red food coloring (3 in Figure 4e). The average pixel intensity in the analyzed area of the detection chamber changed accordingly and complete replenishment of the detection chamber took ∼16 min (Figure 4e). The gradual change of color in the detection chamber, instead of a sharp transition, is due to Taylor dispersion.^53,54^

### Multiplexed Continuous Monitoring of Interleukin-6 and TNFα

**Figure 5:**
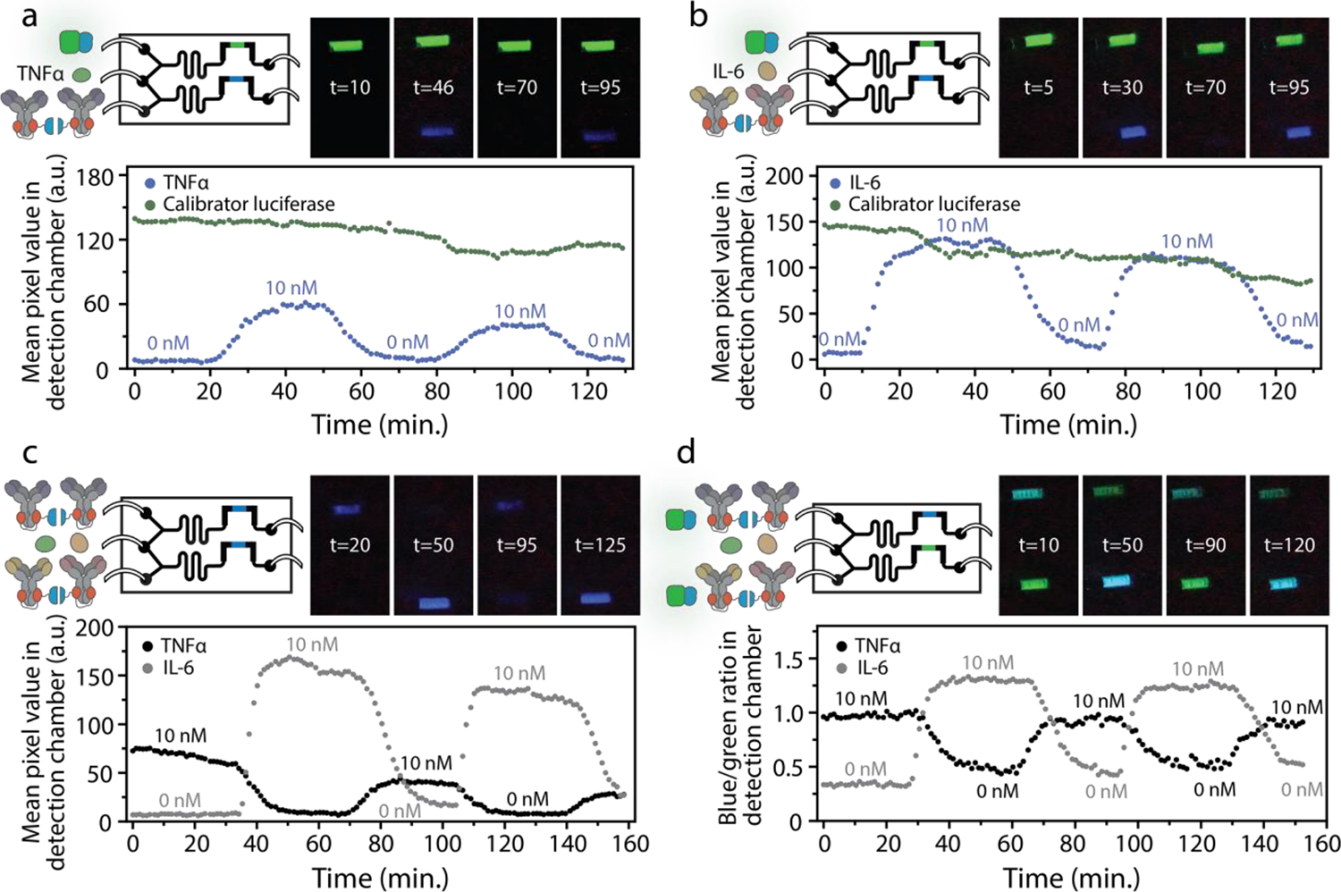
Multiplexed detection of interleukin-6 and TNFα in the dRAPPID continuous monitoring microfluidic chip. (a) Intensiometric continuous detection of TNFα. The calibrator luciferase (60 pM) was added to the upper sensor inlet, TNFα dRAPPID was injected in the lower sensor inlet, and the TNFα concentration, in the analyte inlet, was switched between 0 and 10 nM. (b) Intensiometric continuous detection of IL-6. The green calibrator luciferase (75 pM) was injected in the upper sensor inlet and the IL-6 dRAPPID in the lower inlet. 10 or 0 nM of IL-6 was injected into the analyte inlet, and the corresponding blue light response in the lower detection chamber was measured with a digital camera. (c) Multiplexed intensiometric detection of IL-6 and TNFα. TNFα dRAPPID was added to the upper sensor inlet, and IL-6 dRAPPID was injected into the lower sensor inlet. Both IL-6 and TNFα were added in the sensor inlet, either at 0 or 10 nM, and the responsive change in blue light emission was detected in both detection chambers. (d) Multiplexed ratiometric detection of IL-6 and TNFα. TNFα dRAPPID with 27 pM calibrator and IL-6 dRAPPID with 60 pM calibrator were injected in the upper and lower sensor inlet, respectively. All dRAPPID assays were performed with 1 nM Ab-LB, 10 nM Ab-SB, and 250× diluted substrate in buffer (PBS (pH 7.4), 0.1% (w/v) BSA).

To demonstrate that the continuous monitoring chip, in combination with dRAPPID, can be used to detect changes in concentrations of relevant biomolecules, we next applied the chip to monitor IL-6 and TNFα levels. Using the same flow rates and setup as with the food coloring experiments (Figure 4e), we first injected TNFα as an analyte in the sample inlet and TNFα dRAPPID in the lower sensor inlet of the microfluidic chip. The calibrator luciferase, unresponsive to the analyte concentration, was added to the upper sensor inlet of the chip to determine the effect of the long continuous measurement on the luminescent signal in the detection chambers. We started by adding buffer without TNFα into the chip (0 nM TNFα), resulting in the expected absence of a blue luminescent signal in the lower detection chamber in Figure 5a. Switching to 10 nM TNFα caused an increase in blue signal, due to complex formation between TNFα and the dRAPPID sensor and the consequential complementation of the split NLuc. Switching back to 0 nM TNFα and subsequently increasing the concentration to 10 nM resulted in the disappearance and appearance of the blue signal in the lower detection chamber, respectively, demonstrating the continuous detection of changes in TNFα concentrations. As expected, the green luminescent signal of the calibrator luciferase in the upper detection chamber did not respond to the changes in injected TNFα concentration. A ∼20% decrease in the signal of both the calibrator luciferase and the dRAPPID sensor was observed over time, however, which is likely due to slow auto-oxidation of the NLuc substrate over the course of the experiment. The blue and green signals decrease with a similar slope, enabling the normalization of the blue responsive signal by the green constant signal and thereby eliminating the effect of NLuc substrate degradation (Figure S11a). Similarly, we injected IL-6 and IL-6 dRAPPID in the microfluidic chip and observed the expected change in blue light in response to different IL-6 concentrations (Figure 5b). Normalization of this responsive blue light by the constant green luminescent signal in the upper detection chamber resulted in a more stable signal over time (Figure S11b).

Next, to demonstrate the simultaneous detection of two relevant biomolecules, we added TNFα dRAPPID in the upper sensor inlet and IL-6 dRAPPID in the lower sensor inlet (Figure 5c). The injected sample contained either 0 nM IL-6 and 10 nM TNFα, resulting in a blue signal in the upper detection chamber, or 10 nM IL-6 and 0 nM TNFα, giving a blue signal in the lower detection chamber. Switching between these samples resulted in the expected alternating blue light responses in the detection chambers. Finally, to create a more stable luminescent signal over time and eliminate the effects of NLuc substrate oxidation, we added the calibrator luciferase to the two dRAPPID sensors, enabling multiplexed ratiometric detection of IL-6 and TNFα (Figure 5d). The captured light in the detection chambers changed from blue to green, depending on the concentrations of the target analytes. The plotted blue-to-green ratios were constant over time, as switching back to the same analyte concentration yielded similar blue-to-green ratios. These results demonstrate that the dRAPPID continuous monitoring chip can be used to detect two relevant biomolecules simultaneously and that ratiometric detection yields stable signals over time, enabling sensitive and accurate real-time measurements.

## Conclusions

Herein, we developed a new class of RAPPID sensors, with a low intrinsic background luminescent signal, to detect picomolar concentrations of clinically relevant cytokines. The individual sensor components of the dRAPPID system contain two photo-cross-linkable protein G domains to ensure conjugation to both heavy chains of the target-specific IgG. This format enables more efficient and essentially complete formation of antibody-conjugates at 1:1 stoichiometry and precludes the binding of free protein G luciferase fragments to remaining Fc binding sites, ensuring that the observed luciferase activity only results from analyte-induced NLuc complementation. Subsequently, we applied this dimeric RAPPID to detect cytokines in two different contexts: (1) sampling of concentrations of IL-6 secreted by cells and (2) the multiplexed continuous detection of IL-6 and TNFα concentrations in a microfluidic chip.

Continuous monitoring with the dRAPPID microfluidic chip relies on the continuous inflow of new dRAPPID sensors. Therefore, fast, reversible binding between antibody and target may not be required. This broadens the scope of antibodies that can be used, as high affinity and slow dissociation kinetics do not impede their usage. In the current setup, it takes ∼15 min before the complete detection chamber is refreshed by a new sensor-analyte complex, which might hamper the detection of fluctuations on a very short time scale of minutes. However, a time resolution of 15 min would most likely be sufficient to detect pathophysiological changes in cytokine concentrations. The 15 min delay could be decreased by further reducing the volume of the detection chamber, increasing the flowrates or by introducing active mixing. The continuous sensing platform shows potential to detect biomarkers in in vitro cell models, demonstrated by the minimal difference in ratiometric signal between measurements in buffer and culture medium and the low picomolar sensitivity of the dRAPPID sensors. For measurements in more complex media, such as blood plasma, we expect that samples need to be diluted prior to mixing with the sensor, to decrease possible viscosity-related problems.

The herein-developed continuous sensing platform demonstrates the feasibility of integrating the dRAPPID sensors for point-of-need monitoring. The simple measurement setup, with a light-tight box and a digital camera, and the large variety of available RAPPID sensors allow the detection of a wide panel of biomarkers outside of traditional laboratories. The few continuous monitoring immunoassay-platforms that have been developed so far do not reach picomolar sensitivity or require relatively expensive and complex detection techniques.^33,34,55,56^ In the future, the dRAPPID microfluidic chip can be applied to detect biomarkers in in vitro models, increasing our knowledge about disease progression, therapeutic effectiveness, and organ or cell physiology. Furthermore, the continuous sensing platform can be used in healthcare monitoring, for example, for continuously measuring inflammation markers in the intensive care unit. The detection of sepsis-related biomarkers such as IL-6, procalcitonin, and C-reactive protein, with serum concentrations that can exceed ∼70, ∼600, and **∼**400 pM, respectively, could facilitate early diagnosis of sepsis and predict disease severity.^13,57−59^

## Supporting Information Available

Amino acid and DNA sequences of the pG-d2-LB and SB, SDS-PAGE gels of the expression of pG-d2-LB and pG-d2-SB, fitting of intensiometric data to a thermodynamic model, the photoconjugation set-up, microfluidic mask design, background signal of the different sensor proteins, limit-of-detection measurements, flow rate determination and normalized signals of the continuous monitoring chip (PDF)
