# Supplementary material for: Integrated Bioluminescent Immunoassays for High-Throughput Sampling and Continuous Monitoring of Cytokines
Source: Anal Chem. 2023 May 30;95(23):8922–31. doi: 10.1021/acs.analchem.3c00745 (PMC10267889; doi:10.1021/acs.analchem.3c00745)
Supplement: Supplementary file 1 — ac3c00745_si_001.pdf [file ac3c00745_si_001.pdf]

Supporting information

Integrated bioluminescent immunoassays for high-throughput sampling and continuous monitoring of cytokines

Eva A. van Aalen[ab], Bas J. H. M. Rosier[ab†], Tom Jansen[ab], Simone F. A. Wouters[ab§], Robin T. Vermathen[ab], Harmen J. van der Veer[ab], José Yeste Lozano[c], Sheeza Mughal[c], Juan M. Fernández-Costa[c], Javier Ramón-Azcón[cd], Jaap M. J. den Toonder[be] and Maarten Merkx*[ab]

[a] Laboratory of Chemical Biology, Department of Biomedical Engineering, Eindhoven University of Technology, P.O Box 513, 5600 MB Eindhoven, The Netherlands.
[b] Institute for Complex Molecular Systems, Eindhoven University of Technology, P.O Box 513, 5600 MB Eindhoven, The Netherlands.
[c] Institute for Bioengineering of Catalonia (IBEC), The Barcelona Institute of Science and Technology (BIST), C/ Baldiri Reixac 10-12, Barcelona, E08028, Spain.
[d] Institució Catalana de Recerca i Estudis Avançats (ICREA), Passeig de Lluís Companys, 23,O Barcelona, E08010, Spain.
[e] Microsystems, Department of Mechanical Engineering, Eindhoven University of Technology, P.O Box 513, 5600 MB Eindhoven, The Netherlands.

Content Page

Figure S1	Thermodynamic models	S2-S8
Figure S2	DNA and amino acid sequence of Gx-d2-LB	S9
Figure S3	DNA and amino acid sequence of Gx-d2-SB	S10
Figure S4	Expression and purification of Gx-d2-SB and Gx-d2-LB	S11
Figure S5	The RAPPID photoconjugation set-up	S11
Figure S6	dRAPPID microfluidic chip mask design	S12
Figure S7	Background comparison between dimeric RAPPID and classical RAPPID	S12
Figure S8	Limit-of-detection measurement of the IL-6 dRAPPID	S13
Figure S9	Picture of the dRAPPID microfluidic chip	S13
Figure S10	Homogenization of fluorescent signal in the serpentine channels	S14
Figure S11	Normalized signal of continuous monitoring chip	S15
	Supporting Information references	S15

Thermodynamic models

To elucidate the underlying thermodynamic balances leading to the observed concentration dependent dRAPPID response and to estimate the affinities of the antibodies used, we developed a model of both the IL-6 and the TNFα assays (Figure S1a, S1b).

IL-6 dRAPPID model

In the IL-6 model, Ab-LB (A) and Ab-SB (B) are considered to bind the target analyte (T) in a non-cooperative fashion, hence Ab-LB binds to free analyte with the same affinity ($K_{D,A}$) as to the target already bound by Ab-SB. Upon formation of the ternary ABTi complex, the high local concentration (the effective molarity (EM)) of the split NanoLuc fragments (with affinity $K_{D,N}$) promotes complementation, transitioning to the luminescent ABTa complex (Figure S1a). Although not exhaustive, multiple other potential interactions of the three components (including relevant statistical factors) are included that are assumed to be the most important contributing factors to the observed luminescent signal. In addition to ABTa and double target bound Tatbs and Tbtas complexes, also non-templated split NanoLuc complementation could result in luminescence, hence the total luminescent signal is modelled as the sum of the concentrations of AB, Tab, Tba, ABTa, Tatbs, and Tbtas, multiplied by a constant.

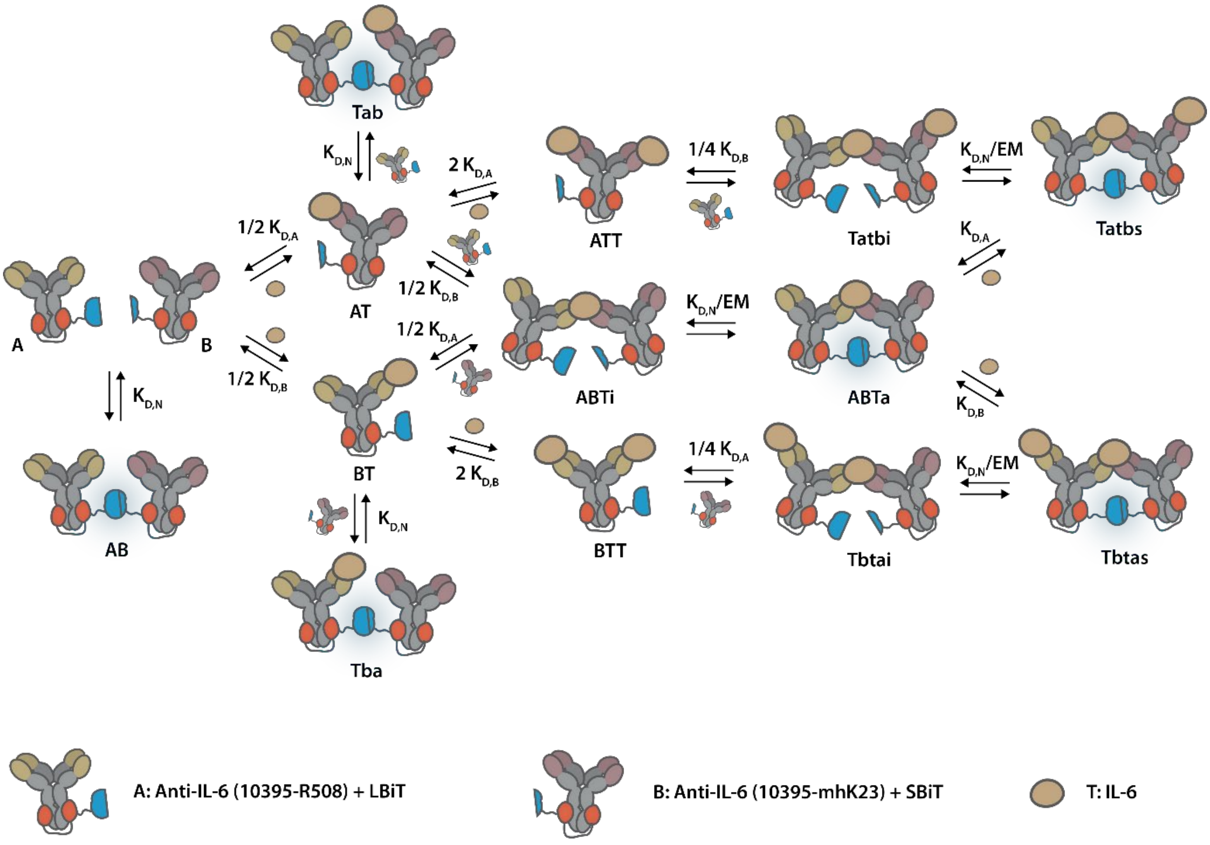

Figure S1a. Model of the thermodynamic interactions in the IL-6 dRAPPID assay.

The model comprises an extended version of the model used in the original RAPPID publication[1] and was implemented using a general framework for equilibrium models developed by Geertjens *et al.*[2], using the following input to build the model in the Python tool:

```
Equations:
A + B = AB; KdN
A + T = TA; 1/2*KdA
```

```
B + T = TB; 1/2*KdB
TB + T = TTB; 2*KdB
TB + A = ABTi; 1/2*KdA
TA + T = TTA; 2*KdA
TA + B = ABTi; 1/2*KdB
ABTi = ABTa; KdN / EM
TB + A = Tba; KdN
TA + B = Tab; KdN
TAT + B = Tatbi; 1/4*KdB
TBT + A = Tbtai; 1/4*KdA
Tatbi = Tatbs; KdN / EM
Tbtai = Tbtas; KdN / EM
ABTa + T = Tatbs; KdA
ABTa + T = Tbtas; KdB

data_mode: custom
custom input: constant * (ABTa + Tatbs + Tbtas + Tba + Tab + AB)
```

From this, a model was generated in the framework, which is further detailed below for completeness:

The equilibrium concentrations of the dependent species can be determined based on the concentrations of the independent species and the corresponding equilibrium constant, using the following relations:

```
AB: A*B/KdN
AT: 2*A*T/KdA
BT: 2*B*T/KdB
BTT: B*T**2/KdB**2
ABTi: 4*A*B*T/(KdA*KdB)
ATT: A*T**2/KdA**2
ABTa: 4*A*B*EM*T/(KdA*KdB*KdN)
Tba: 2*A*B*T/(KdB*KdN)
Tab: 2*A*B*T/(KdA*KdN)
Tatbi: 4*A*B*T**2/(KdA**2*KdB)
Tbtai: 4*A*B*T**2/(KdA*KdB**2)
Tatbs: 4*A*B*EM*T**2/(KdA**2*KdB*KdN)
Tbtas: 4*A*B*EM*T**2/(KdA*KdB**2*KdN)
```

The mass balance of the independent species in terms of free and complexed forms:

```
B_tot: AB + ABTa + ABTi + B + BT + BTT + Tab + Tatbi + Tatbs + Tba + Tbtai
+ Tbtas
T_tot: ABTa + ABTi + AT + 2*ATT + BT + 2*BTT + T + Tab + 2*Tatbi + 2*Tatbs
+ Tba + 2*Tbtai + 2*Tbtas
A_tot: A + AB + ABTa + ABTi + AT + ATT + Tab + Tatbi + Tatbs + Tba + Tbtai
+ Tbtas
```

Substituting the relations above in the mass balance equations yields:

```
B_tot = 4*A*B*EM*T/(KdA*KdB*KdN) + 4*A*B*EM*T**2/(KdA*KdB**2*KdN) +
4*A*B*EM*T**2/(KdA**2*KdB*KdN) + A*B/KdN + 2*A*B*T/(KdB*KdN) +
2*A*B*T/(KdA*KdN) + 4*A*B*T/(KdA*KdB) + 4*A*B*T**2/(KdA*KdB**2) +
4*A*B*T**2/(KdA**2*KdB) + B + 2*B*T/KdB + B*T**2/KdB**2
T_tot = 4*A*B*EM*T/(KdA*KdB*KdN) + 8*A*B*EM*T**2/(KdA*KdB**2*KdN) +
8*A*B*EM*T**2/(KdA**2*KdB*KdN) + 2*A*B*T/(KdB*KdN) + 2*A*B*T/(KdA*KdN) +
4*A*B*T/(KdA*KdB) + 8*A*B*T**2/(KdA*KdB**2) + 8*A*B*T**2/(KdA**2*KdB) +
2*A*T/KdA + 2*A*T**2/KdA**2 + 2*B*T/KdB + 2*B*T**2/KdB**2 + T
A_tot = 4*A*B*EM*T/(KdA*KdB*KdN) + 4*A*B*EM*T**2/(KdA*KdB**2*KdN) +
4*A*B*EM*T**2/(KdA**2*KdB*KdN) + A*B/KdN + 2*A*B*T/(KdB*KdN) +
```

```
2*A*B*T/(KdA*KdN) + 4*A*B*T/(KdA*KdB) + 4*A*B*T**2/(KdA*KdB**2) +
4*A*B*T**2/(KdA**2*KdB) + A + 2*A*T/KdA + A*T**2/KdA**2
```

Finally, these equations were rewritten to equal zero, and divided by the total concentrations on both sides in order to reach a solution faster during solving:

```
B: (4*A*B*EM*T/(KdA*KdB*KdN) + 4*A*B*EM*T**2/(KdA*KdB**2*KdN) +
4*A*B*EM*T**2/(KdA**2*KdB*KdN) + A*B/KdN + 2*A*B*T/(KdB*KdN) +
2*A*B*T/(KdA*KdN) + 4*A*B*T/(KdA*KdB) + 4*A*B*T**2/(KdA*KdB**2) +
4*A*B*T**2/(KdA**2*KdB) + B + 2*B*T/KdB + B*T**2/KdB**2 - B_tot) / B_tot =
0
T: (4*A*B*EM*T/(KdA*KdB*KdN) + 8*A*B*EM*T**2/(KdA*KdB**2*KdN) +
8*A*B*EM*T**2/(KdA**2*KdB*KdN) + 2*A*B*T/(KdB*KdN) + 2*A*B*T/(KdA*KdN) +
4*A*B*T/(KdA*KdB) + 8*A*B*T**2/(KdA*KdB**2) + 8*A*B*T**2/(KdA**2*KdB) +
2*A*T/KdA + 2*A*T**2/KdA**2 + 2*B*T/KdB + 2*B*T**2/KdB**2 + T - T_tot) /
T_tot = 0
A: (4*A*B*EM*T/(KdA*KdB*KdN) + 4*A*B*EM*T**2/(KdA*KdB**2*KdN) +
4*A*B*EM*T**2/(KdA**2*KdB*KdN) + A*B/KdN + 2*A*B*T/(KdB*KdN) +
2*A*B*T/(KdA*KdN) + 4*A*B*T/(KdA*KdB) + 4*A*B*T**2/(KdA*KdB**2) +
4*A*B*T**2/(KdA**2*KdB) + A + 2*A*T/KdA + A*T**2/KdA**2 - A_tot) / A_tot =
0
```

This model was fitted to the data presented in Figure 2a using $K_{D,N}$ = 2.5E-6 M as known parameter[3]. The affinities of the antibodies for IL-6 are unknown; for an initial estimate $K_{D.A}$ and $K_{D,B}$ were set to 10E-9 M. The effective molarity of the split NanoLuc fragments (EM) depends on the length and flexibility of the linkers connecting them to the antibodies, as well as the distance that has to be bridged by the linkers for luciferase complementation. As this distance strongly depends on the unknown binding geometry of the two antibodies to the IL-6, an EM is difficult to predict using a wormlike chain model[4,5]. As a rough initial estimate EM was set to 1E-6 M. For the factor converting luminescent entity concentrations to luminescence signal intensity, the initial value was set as 'constant' = 1E+16. The following parameter estimates were obtained (fitted model shown in Figure 2a):

```
EM: 7.200e-06
KdA: 1.204e-07
KdB: 2.301e-09
constant: 1.870e+16

Root Mean Squared Error = 1.72e+05
```
R^2 = 0,995

TNFα dRAPPID model

Similarly, we developed a thermodynamic model of the TNFα dRAPPID assay (Figure S1b). Also in the TNFα model, Ab-LB (L) and Ab-SB (S) are considered to bind the target analyte (T) in a non-cooperative fashion, hence Ab-LB binds to free analyte with the same affinity ($K_{D,Ab}$) as to the target already bound by Ab-SB. Since TNFα is a trimeric protein, both Ab-LB and Ab-SB are made up of the same antibody in the TNFα dRAPPID assay, and hence a single target-binding affinity ($K_{D,Ab}$) is used for the interactions of both components. Moreover, the three corresponding epitopes per TNFα trimer allow for the formation of additional multimeric complexes. A non-exhaustive model was set up including multiple of such potential interactions of the three components (including relevant statistical factors) that are assumed to be the most important contributing factors to the observed luminescent signal, including up to 4 components per complex. The total luminescent signal is modelled as the sum of the concentrations of LS, Tsl, Tls, Tstla, Tltsa, LSTa, LSSTa and LLSTa multiplied by a constant.

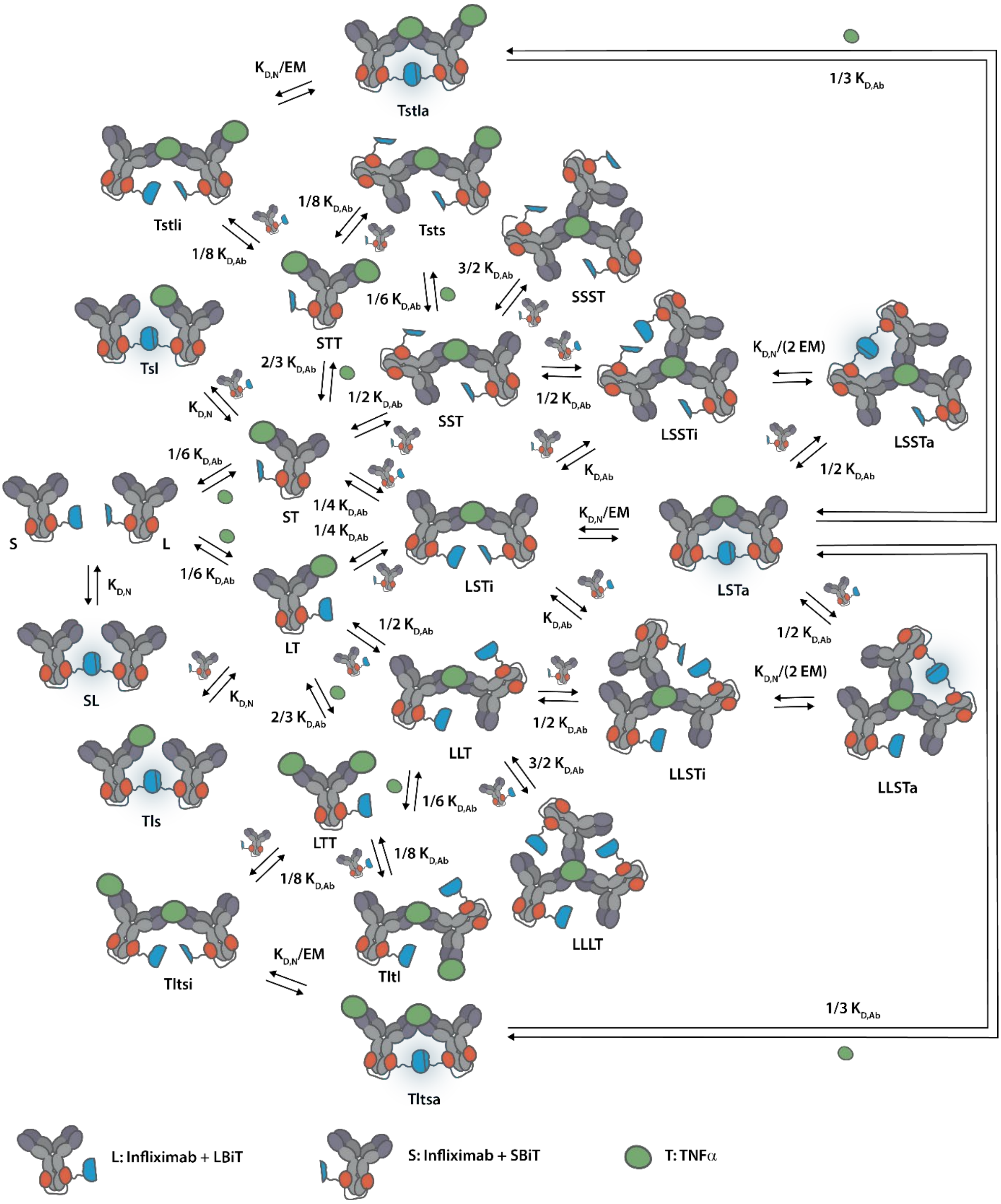

Figure S1b. Model of the thermodynamic interactions in the TNFα dRAPPID assay.

Similar to the IL-6 dRAPPID model, the TNFα dRAPPID model was implemented using the general framework for equilibrium models developed by Geertjens *et al.*[2], using the following input to build the model in the Python tool:

```
Equations:
L + S = LS; KdN
S + T = ST; 1/6*KdAb
L + T = LT; 1/6*KdAb
ST + T = STT; 2/3*KdAb
```

```
ST + L = LSTi; 1/4*KdAb
LT + T = LTT; 2/3*KdAb
LT + S = LSTi; 1/4*KdAb
LSTi = LSTa; KdN / EM
ST + L = Tsl; KdN
LT + S = Tls; KdN
STT + L = Tstli; 1/8*KdAb
LTT + S = Tltsi; 1/8*KdAb
Tstli = Tstla; KdN / EM
Tltsi = Tltsa; KdN / EM
LSTa + T = Tstla; 1/3*KdAb
LSTa + T = Tltsa; 1/3*KdAb
LSTi + S = LSSTi; KdAb
LSTi + L = LLSTi; KdAb
LSSTi = LSSTa; 1/2*KdN / EM
LLSTi = LLSTa; 1/2*KdN / EM
LSTa + S = LSSTa; 1/2*KdAb
LSTa + L = LLSTa; 1/2*KdAb
LT + L = LLT; 2/4*KdAb
ST + S = SST; 2/4*KdAb
LLT + L = LLLT; 3/2*KdAb
SST + S = SSST; 3/2*KdAb
LLT + S = LLSTi; 1/2*KdAb
SST + L = LSSTi; 1/2*KdAb
Tstli + T = Tstlti; 1/3*KdAb
Tltsi + T = Tltsti; 1/3*KdAb
Tstlti = Tstlta; KdN / EM
Tltsti = Tltsta; KdN / EM
Tstla + T = Tstlta; 1/3*KdAb
Tltsa + T = Tltsta; 1/3*KdAb
STT + S = Tsts; 1/8*KdAb
LTT + L = Tltl; 1/8*KdAb
SST + T = Tsts; 1/6*KdAb
LLT + T = Tltl; 1/6*KdAb

data_mode: custom
custom input: constant * (Tsl + Tls + LS + LSTa + Tstla + Tltsa + LSSTa +
LLSTa)
```

From this, a model was generated in the framework, which is further detailed below for completeness:

The equilibrium concentrations of the dependent species can be determined based on the concentrations of the independent species and the corresponding equilibrium constant, using the following relations:

```
LS: L*S/KdN
ST: 6*S*T/KdAb
LT: 6*L*T/KdAb
STT: 9*S*T**2/KdAb**2
LSTi: 24*L*S*T/KdAb**2
LTT: 9*L*T**2/KdAb**2
LSTa: 24*EM*L*S*T/(KdAb**2*KdN)
Tsl: 6*L*S*T/(KdAb*KdN)
Tls: 6*L*S*T/(KdAb*KdN)
Tstli: 72*L*S*T**2/KdAb**3
Tltsi: 72*L*S*T**2/KdAb**3
Tstla: 72*EM*L*S*T**2/(KdAb**3*KdN)
Tltsa: 72*EM*L*S*T**2/(KdAb**3*KdN)
LSSTi: 24*L*S**2*T/KdAb**3
LLSTi: 24*L**2*S*T/KdAb**3
```

```
LSSTa: 48*EM*L*S**2*T/(KdAb**3*KdN)
LLSTa: 48*EM*L**2*S*T/(KdAb**3*KdN)
LLT: 12*L**2*T/KdAb**2
SST: 12*S**2*T/KdAb**2
LLLT: 8*L**3*T/KdAb**3
SSST: 8*S**3*T/KdAb**3
Tstlti: 216*L*S*T**3/KdAb**4
Tltsti: 216*L*S*T**3/KdAb**4
Tstlta: 216*EM*L*S*T**3/(KdAb**4*KdN)
Tltsta: 216*EM*L*S*T**3/(KdAb**4*KdN)
Tsts: 72*S**2*T**2/KdAb**3
Tltl: 72*L**2*T**2/KdAb**3
```

The mass balance of the independent species in terms of free and complexed forms:

```
S_tot: LLSTa + LLSTi + LS + 2*LSSTa + 2*LSSTi + LSTa + LSTi + S + 3*SSST +
2*SST + ST + STT + Tls + Tltsa + Tltsi + Tltsta + Tltsti + Tsl + Tstla +
Tstli + Tstlta + Tstlti + 2*Tsts
L_tot: L + 3*LLLT + 2*LLSTa + 2*LLSTi + 2*LLT + LS + LSSTa + LSSTi + LSTa +
LSTi + LT + LTT + Tls + 2*Tltl + Tltsa + Tltsi + Tltsta + Tltsti + Tsl +
Tstla + Tstli + Tstlta + Tstlti
T_tot: LLLT + LLSTa + LLSTi + LLT + LSSTa + LSSTi + LSTa + LSTi + LT +
2*LTT + SSST + SST + ST + 2*STT + T + Tls + 2*Tltl + 2*Tltsa + 2*Tltsi +
3*Tltsta + 3*Tltsti + Tsl + 2*Tstla + 2*Tstli + 3*Tstlta + 3*Tstlti +
2*Tsts
```

Substituting the relations above in the mass balance equations yields:

```
S_tot = 24*EM*L*S*T/(KdAb**2*KdN) + 48*EM*L**2*S*T/(KdAb**3*KdN) +
96*EM*L*S**2*T/(KdAb**3*KdN) + 144*EM*L*S*T**2/(KdAb**3*KdN) +
432*EM*L*S*T**3/(KdAb**4*KdN) + S  + L*S/KdN + 6*S*T/KdAb +
12*L*S*T/(KdAb*KdN) + 24*L*S*T/KdAb**2 + 24*S**2*T/KdAb**2 +
9*S*T**2/KdAb**2 + 24*L**2*S*T/KdAb**3 + 48*L*S**2*T/KdAb**3 +
144*L*S*T**2/KdAb**3 + 24*S**3*T/KdAb**3 + 144*S**2*T**2/KdAb**3 +
432*L*S*T**3/KdAb**4
L_tot = 24*EM*L*S*T/(KdAb**2*KdN) + 96*EM*L**2*S*T/(KdAb**3*KdN) +
48*EM*L*S**2*T/(KdAb**3*KdN) + 144*EM*L*S*T**2/(KdAb**3*KdN) +
432*EM*L*S*T**3/(KdAb**4*KdN) + L  + L*S/KdN + 6*L*T/KdAb +
12*L*S*T/(KdAb*KdN) + 24*L**2*T/KdAb**2 + 24*L*S*T/KdAb**2 +
9*L*T**2/KdAb**2 + 24*L**3*T/KdAb**3 + 48*L**2*S*T/KdAb**3 +
144*L**2*T**2/KdAb**3 + 24*L*S**2*T/KdAb**3 + 144*L*S*T**2/KdAb**3 +
432*L*S*T**3/KdAb**4
T_tot = 24*EM*L*S*T/(KdAb**2*KdN) + 48*EM*L**2*S*T/(KdAb**3*KdN) +
48*EM*L*S**2*T/(KdAb**3*KdN) + 288*EM*L*S*T**2/(KdAb**3*KdN) +
1296*EM*L*S*T**3/(KdAb**4*KdN) + T  + 6*L*T/KdAb + 6*S*T/KdAb +
12*L*S*T/(KdAb*KdN) + 12*L**2*T/KdAb**2 + 24*L*S*T/KdAb**2 +
18*L*T**2/KdAb**2 + 12*S**2*T/KdAb**2 + 18*S*T**2/KdAb**2 +
8*L**3*T/KdAb**3 + 24*L**2*S*T/KdAb**3 + 144*L**2*T**2/KdAb**3 +
24*L*S**2*T/KdAb**3 + 288*L*S*T**2/KdAb**3 + 8*S**3*T/KdAb**3 +
144*S**2*T**2/KdAb**3 + 1296*L*S*T**3/KdAb**4
```

Finally, these equations were rewritten to equal zero, and divided by the total concentrations on both sides in order to reach a solution faster during solving:

```
S: (24*EM*L*S*T/(KdAb**2*KdN) + 48*EM*L**2*S*T/(KdAb**3*KdN) +
96*EM*L*S**2*T/(KdAb**3*KdN) + 144*EM*L*S*T**2/(KdAb**3*KdN) +
432*EM*L*S*T**3/(KdAb**4*KdN) + S - S_tot + L*S/KdN + 6*S*T/KdAb +
12*L*S*T/(KdAb*KdN) + 24*L*S*T/KdAb**2 + 24*S**2*T/KdAb**2 +
9*S*T**2/KdAb**2 + 24*L**2*S*T/KdAb**3 + 48*L*S**2*T/KdAb**3 +
```

```
144*L*S*T**2/KdAb**3 + 24*S**3*T/KdAb**3 + 144*S**2*T**2/KdAb**3 +
432*L*S*T**3/KdAb**4) / S_tot = 0
L: (24*EM*L*S*T/(KdAb**2*KdN) + 96*EM*L**2*S*T/(KdAb**3*KdN) +
48*EM*L*S**2*T/(KdAb**3*KdN) + 144*EM*L*S*T**2/(KdAb**3*KdN) +
432*EM*L*S*T**3/(KdAb**4*KdN) + L - L_tot + L*S/KdN + 6*L*T/KdAb +
12*L*S*T/(KdAb*KdN) + 24*L**2*T/KdAb**2 + 24*L*S*T/KdAb**2 +
9*L*T**2/KdAb**2 + 24*L**3*T/KdAb**3 + 48*L**2*S*T/KdAb**3 +
144*L**2*T**2/KdAb**3 + 24*L*S**2*T/KdAb**3 + 144*L*S*T**2/KdAb**3 +
432*L*S*T**3/KdAb**4) / L_tot = 0
T: (24*EM*L*S*T/(KdAb**2*KdN) + 48*EM*L**2*S*T/(KdAb**3*KdN) +
48*EM*L*S**2*T/(KdAb**3*KdN) + 288*EM*L*S*T**2/(KdAb**3*KdN) +
1296*EM*L*S*T**3/(KdAb**4*KdN) + T - T_tot + 6*L*T/KdAb + 6*S*T/KdAb +
12*L*S*T/(KdAb*KdN) + 12*L**2*T/KdAb**2 + 24*L*S*T/KdAb**2 +
18*L*T**2/KdAb**2 + 12*S**2*T/KdAb**2 + 18*S*T**2/KdAb**2 +
8*L**3*T/KdAb**3 + 24*L**2*S*T/KdAb**3 + 144*L**2*T**2/KdAb**3 +
24*L*S**2*T/KdAb**3 + 288*L*S*T**2/KdAb**3 + 8*S**3*T/KdAb**3 +
144*S**2*T**2/KdAb**3 + 1296*L*S*T**3/KdAb**4) / T_tot = 0
```

This model was fitted to the data presented in Figure 2e using $K_{D,N}$ = 2.5E-6 M as known parameter[3]. The affinity of Infliximab for TNFα is unknown; for an initial estimate $K_{D.Ab}$ was set to 100E-9 M. Similar to the IL-6 case, an EM is difficult to predict using a wormlike chain model[4,5] as the distance to be bridged by the linkers connecting the antibodies to the split NanoLuc fragments strongly depends on the unknown binding geometry of the antibodies to the TNFα. As a rough initial estimate EM was set to 1E-6 M. For the factor converting luminescent entity concentrations to luminescence signal intensity, the initial value was set as 'constant' = 1E+16. The following parameter estimates were obtained (fitted model shown in Figure 2e):

```
EM: 3.206e-06
KdAb: 1.554e-07
constant: 2.547e+16

Root Mean Squared Error = 3.30e+05
```
R^2 = 0,947

```
M  G  W  S  H  P  Q  F  E  K  G  G  S  M  T  F  K  L  I  I  N  G  K  T  L
ATGGGCTGGAGCCATCCGCAGTTTGAAAAAGGTGGTAGCATGACATTTAAACTGATTATCAACGGCAAAACTTTA
1        10        20        30        40        50        60        70
K  G  E  I  T  I  E  A  V  D  A  *  E  A  E  K  I  F  K  Q  Y  A  N  D  Y
AAGGGAGAGATCACAATAGAAGCGGTGGATGCTTAGGAGGCGGAGAAGATTTTTAAGCAGTATGCAAATGATTAT
76   80        90        100       110       120       130       140
G  I  D  G  E  W  T  Y  D  D  A  T  K  T  F  T  V  T  E  E  F  T  G  G  S
GGAATTGATGGTGAATGGACTTATGACGACGCAACTAAAACTTTCACGGTAACAGAAGAATTTACAGGAGGTTCG
151       160       170       180       190       200       210
G  G  S  G  G  S  G  G  S  G  G  S  G  G  S  G  E  F  A  E  A  A  A  K  E
GGTGGGTCGGGAGGTTCTGGCGGCTCTGGAGGAAGTGGTGGTAGCGGTGAATTCGCCGAAGCAGCCGCTAAAGAA
226  230       240       250       260       270       280       290
A  A  A  K  E  A  A  A  K  E  A  A  A  K  E  A  A  A  K  E  A  A  A  K  A
GCCGCAGCAAAGGAAGCCGCGGCCAAGGAGGCAGCCGCAAAAGAGGCCGCGGCGAAGGAAGCAGCAGCCAAGGCA
301       310       320       330       340       350       360
E  F  G  G  S  G  G  S  G  G  S  G  G  S  G  G  S  G  G  S  G  G  T  M  T
GAATTCGGGGGTAGCGGCGGCTCGGGGGGTAGTGGTGGAAGCGGGGGTTCAGGCGGTTCTGGGGGTACCATGACA
376  380       390       400       410       420       430       440
F  K  L  I  I  N  G  K  T  L  K  G  E  I  T  I  E  A  V  D  A  *  E  A  E
TTTAAACTGATAATCAACGGCAAAACCTTAAAAGGGGAGATCACAATTGAGGCAGTCGATGCCTAGGAAGCCGAG
451       460       470       480       490       500       510
K  I  F  K  Q  Y  A  N  D  Y  G  I  D  G  E  W  T  Y  D  D  A  T  K  T  F
AAAATCTTTAAACAATATGCTAATGATTATGGTATTGACGGAGAATGGACGTATGACGATGCGACAAAAACTTTC
526  530       540       550       560       570       580       590
T  V  T  E  L  T  G  G  S  G  G  S  G  G  S  G  G  S  G  G  S  G  G  S  G
ACCGTAACTGAGCTCACAGGAGGTTCGGGTGGGTCGGGAGGTTCTGGCGGCTCTGGAGGAAGTGGTGGTAGCGGT
601       610       620       630       640       650       660
E  F  A  E  A  A  A  K  E  A  A  A  K  E  A  A  A  K  E  A  A  A  K  E  A
GAATTCGCCGAAGCAGCCGCTAAAGAAGCCGCAGCAAAGGAAGCCGCGGCCAAGGAGGCAGCCGCAAAAGAGGCC
676  680       690       700       710       720       730       740
A  A  K  E  A  A  A  K  A  E  F  G  G  S  G  G  S  G  G  S  G  G  S  G  G
GCGGCGAAGGAAGCAGCAGCCAAGGCAGAATTCGGGGGTAGCGGCGGCTCGGGGGGTAGTGGTGGAAGCGGGGGT
751       760       770       780       790       800       810
S  G  G  S  G  G  T  V  F  T  L  E  D  F  V  G  D  W  E  Q  T  A  A  Y  N
TCAGGCGGTTCTGGGGGTACCGTCTTCACACTCGAAGATTTCGTTGGGGACTGGGAACAGACAGCCGCCTACAAC
826  830       840       850       860       870       880       890
L  D  Q  V  L  E  Q  G  G  V  S  S  L  L  Q  N  L  A  V  S  V  T  P  I  Q
CTGGACCAAGTCCTTGAACAGGGAGGTGTGTCCAGTTTGCTGCAGAATCTCGCCGTGTCCGTAACTCCGATCCAA
901       910       920       930       940       950       960
R  I  V  R  S  G  E  N  A  L  K  I  D  I  H  V  I  I  P  Y  E  G  L  S  A
AGGATTGTCCGGAGCGGTGAAAATGCCCTGAAGATCGACATCCATGTCATCATCCCGTATGAAGGTCTGAGCGCC
976  980       990       1000      1010      1020      1030      1040
D  Q  M  A  Q  I  E  E  V  F  K  V  V  Y  P  V  D  D  H  H  F  K  V  I  L
GACCAAATGGCCCAGATCGAAGAGGTGTTTAAGGTGGTGTACCCTGTGGATGATCATCACTTTAAGGTGATCCTG
1051          1060      1070      1080      1090      1100      1110
P  Y  G  T  L  V  I  D  G  V  T  P  N  M  L  N  Y  F  G  R  P  Y  E  G  I
CCCTATGGCACACTGGTAATCGACGGGGTTACGCCGAACATGCTGAACTATTTCGGACGGCCGTATGAAGGCATC
1126    1130      1140      1150      1160      1170      1180      1190
A  V  F  D  G  K  K  I  T  V  T  G  T  L  W  N  G  N  K  I  I  D  E  R  L
GCCGTGTTCGACGGCAAAAAGATCACTGTAACAGGGACCCTGTGGAACGGCAACAAAATTATCGACGAGCGCCTG
1201          1210      1220      1230      1240      1250      1260
I  T  P  D  G  S  M  L  F  R  V  T  I  N  S  G  G  S  H  H  H  H  H  H  *
ATCACCCCCGACGGCTCCATGCTGTTCCGAGTAACCATCAACAGCGGAGGTTCCCACCACCACCATCACCACTAA
1276    1280      1290      1300      1310      1320      1330      1340
```

Figure S2. DNA and amino acid sequence of Gx-d2-LB. Strep tag (pink), protein G domain (gray), amber stop codon (yellow), LargeBiT (blue) and His-tag (green).

```
M  G  W  S  H  P  Q  F  E  K  G  G  S  M  T  F  K  L  I  I  N  G  K  T  L
ATGGGCTGGAGCCATCCGCAGTTTGAAAAAGGTGGTAGCATGACATTTAAACTGATTATCAACGGCAAAACTTTA
1          10         20         30         40         50         60         70
K  G  E  I  T  I  E  A  V  D  A  *  E  A  E  K  I  F  K  Q  Y  A  N  D  Y
AAGGGAGAGATCACAATAGAAGCGGTGGATGCTTAGGAGGCGGAGAAGATTTTTAAGCAGTATGCAAATGATTAT
76     80         90         100        110        120        130        140
G  I  D  G  E  W  T  Y  D  D  A  T  K  T  F  T  V  T  E  E  F  T  G  G  S
GGAATTGATGGTGAATGGACTTATGACGACGCAACTAAAACTTTCACGGTAACAGAAGAATTTACAGGAGGTTCG
151          160        170        180        190        200        210
G  G  S  G  G  S  G  G  S  G  G  S  G  G  S  G  E  F  A  E  A  A  A  K  E
GGTGGGTCGGGAGGTTCTGGCGGCTCTGGAGGAAGTGGTGGTAGCGGTGAATTCGCCGAAGCAGCCGCTAAAGAA
226     230        240        250        260        270        280        290
A  A  A  K  E  A  A  A  K  E  A  A  A  K  E  A  A  A  K  E  A  A  A  K  A
GCCGCAGCAAAGGAAGCCGCGGCCAAGGAGGCAGCCGCAAAAGAGGCCGCGGCGAAGGAAGCAGCAGCCAAGGCA
301          310        320        330        340        350        360
E  F  G  G  S  G  G  S  G  G  S  G  G  S  G  G  S  G  G  S  G  G  T  M  T
GAATTCGGGGGTAGCGGCGGCTCGGGGGGTAGTGGTGGAAGCGGGGGTTCAGGCGGTTCTGGGGGTACCATGACA
376     380        390        400        410        420        430        440
F  K  L  I  I  N  G  K  T  L  K  G  E  I  T  I  E  A  V  D  A  *  E  A  E
TTTAAACTGATAATCAACGGCAAAACCTTAAAAGGGGAGATCACAATTGAGGCAGTCGATGCCTAGGAAGCCGAG
451          460        470        480        490        500        510
K  I  F  K  Q  Y  A  N  D  Y  G  I  D  G  E  W  T  Y  D  D  A  T  K  T  F
AAAATCTTTAAACAATATGCTAATGATTATGGTATTGACGGAGAATGGACGTATGACGATGCGACAAAAACTTTC
526     530        540        550        560        570        580        590
T  V  T  E  L  T  G  G  S  G  G  S  G  G  S  G  G  S  G  G  S  G  G  S  G
ACCGTAACTGAGCTCACAGGAGGTTCGGGTGGGTCGGGAGGTTCTGGCGGCTCTGGAGGAAGTGGTGGTAGCGGT
601          610        620        630        640        650        660
E  F  A  E  A  A  A  K  E  A  A  A  K  E  A  A  A  K  E  A  A  A  K  E  A
GAATTCGCCGAAGCAGCCGCTAAAGAAGCCGCAGCAAAGGAAGCCGCGGCCAAGGAGGCAGCCGCAAAAGAGGCC
676     680        690        700        710        720        730        740
A  A  K  E  A  A  A  K  A  E  F  G  G  S  G  G  S  G  G  S  G  G  S  G  G
GCGGCGAAGGAAGCAGCAGCCAAGGCAGAATTCGGGGGTAGCGGCGGCTCGGGGGGTAGTGGTGGAAGCGGGGGT
751          760        770        780        790        800        810
S  G  G  S  G  G  T  V  T  G  Y  R  L  F  E  K  E  S  G  G  S  H  H  H  H
TCAGGCGGTTCTGGGGGTACCGTTACCGGCTATCGTCTGTTTGAAAAAGAGAGCGGCGGTTCACATCATCATCAC
826     830        840        850        860        870        880        890
H  H  *
CACCATTAA
901
```

Figure S3. DNA and amino acid sequence of Gx-d2-SB. Strep tag (pink), protein G domain (gray), amber stop codon (yellow), SmallBiT (blue) and His-tag (green).

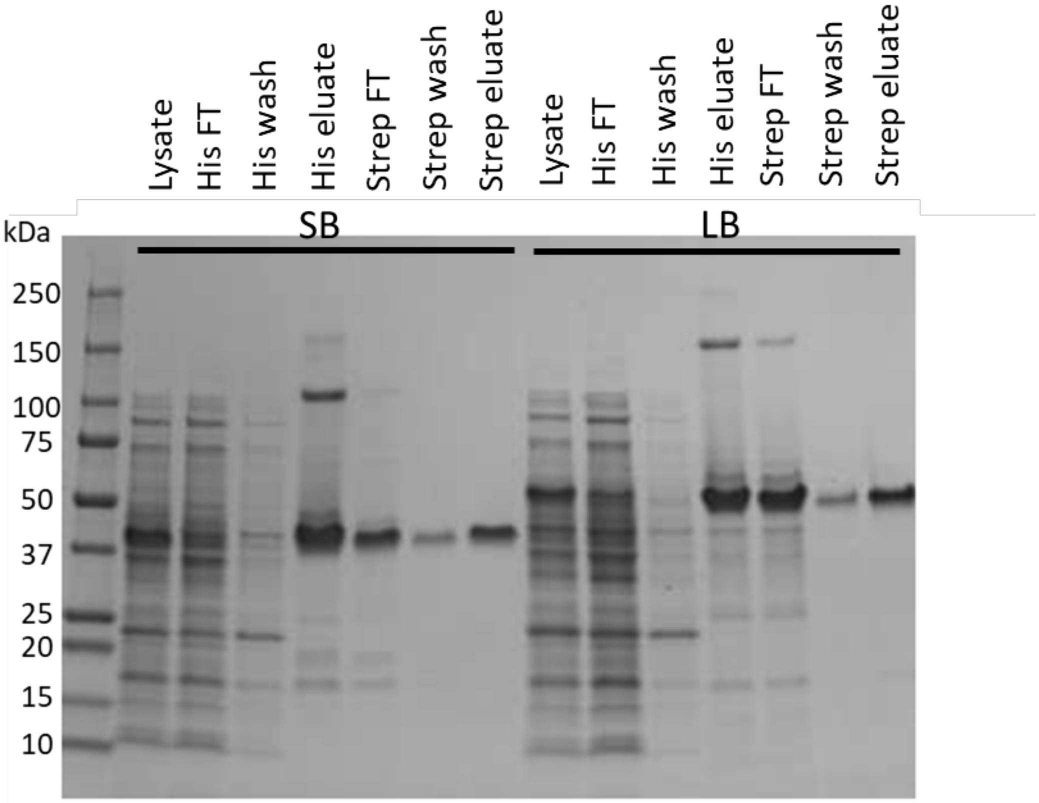

Figure S4. Expression and purification of Gx-d2-SB and Gx-d2-LB. Reducing SDS-PAGE (4-20%) analysis of Gx-d2-SB (SB in gel) and Gx-d2-LB (LB in gel) after expression in *E. coli* and purification using Ni^{2+} affinity chromatography and Strep-Tactin chromatography (FT = flow through).

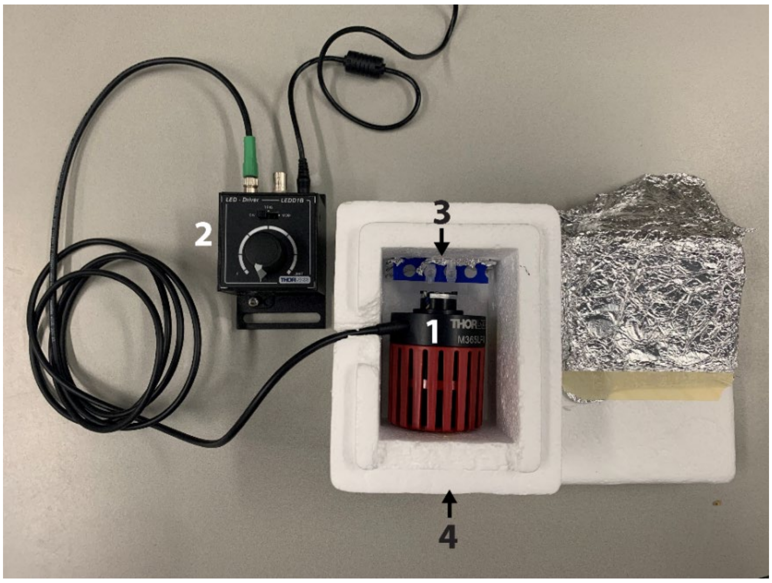

Figure S5. The RAPPID photoconjugation set-up. 1) Thorlabs M365LP1 (λ = 365nm) UV-lamp, 2) Thorlabs LEDD1B T-Cube LED Driver, 3) 0.2 mL PCR tubes with dRAPPID sensor components and antibody in PBS (pH 7.4) and 4) Styrofoam box. Photo-conjugations were performed with 1 μM antibody and 2 μM Gx-d2-SB or Gx-d2-LB and were irradiated for 15 minutes.

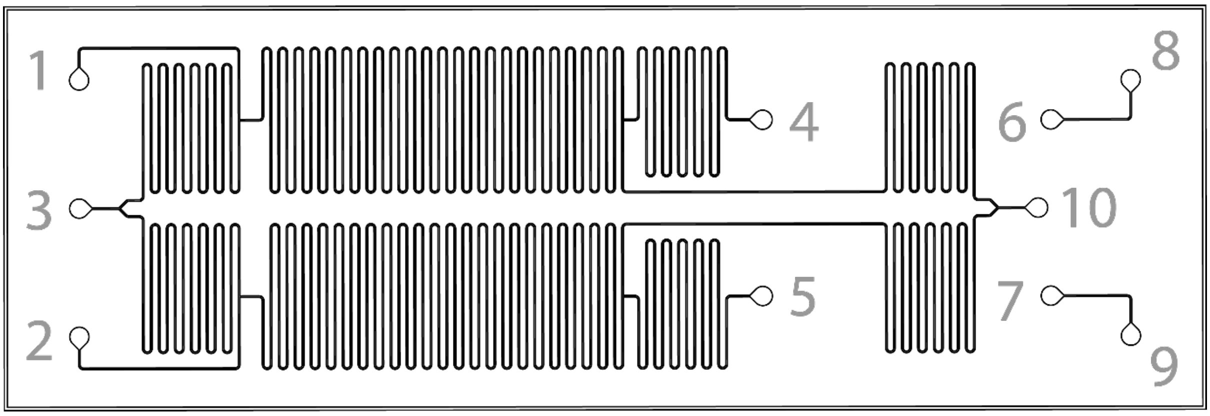

Figure S6. dRAPPID microfluidic chip mask design. The mask was designed using AutoCAD software and was subsequently used to fabricate the SU-8 master wafer. 1) RAPPID sensor inlet 1, 2) RAPPID sensor inlet 2, 3) analyte inlet, 4), 5), 6) and 7) inlets for the incorporation of the detection chambers, 8) outlet 1, 9) outlet 2 and 10) NLuc substrate inlet.

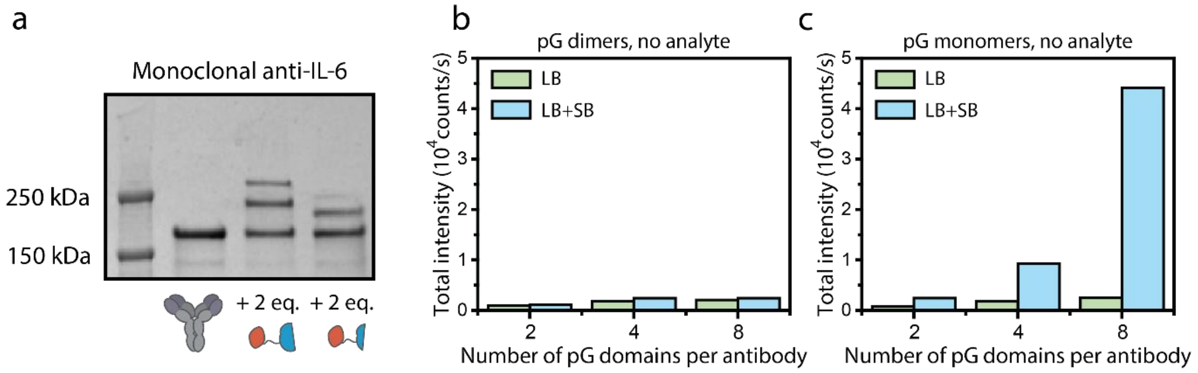

Figure S7. Comparison of background activity for dimeric IL-6 dRAPPID and classical IL-6 RAPPID. (a) Non-reduced SDS-PAGE (4-20%) analysis of the photoconjugation of anti-IL-6 to monomeric pG-LB or pG-SB. Non-conjugated, once-conjugated and twice-conjugated species can be observed. Non-conjugated and once-conjugated species will contribute to undesired background luminescence, due to the binding of a protein G adapter with the complementary fragment of split NLuc. (b) Background luminescent signal of the dimeric protein G adapters (without analyte present). Background luminescence is mainly due to residual luminescence of LB. Increasing the amount of pG dimer domains per antibody resulted in a minimal increase in background luminescence because both heavy chains of one antibody are already occupied with a protein G adapter, preventing the binding of a complementary fragment of split NLuc. (c) Background luminescent signal of the monomeric protein G adapters (without analyte present). Background luminescence is mainly due to LB-SB binding. Increasing the number of pG-LB or SB per antibody greatly increased background signal, due to binding of the complementary fragment of split NLuc to the free heavy chain of the antibody, enabling the reconstitution of NLuc. Experiments in (b) and (c) were done with 0.1 nM anti-IL-6-LB and 1 nM anti-IL-6-SB in buffer (PBS (pH 7.4), 0.1% (w/v) BSA).

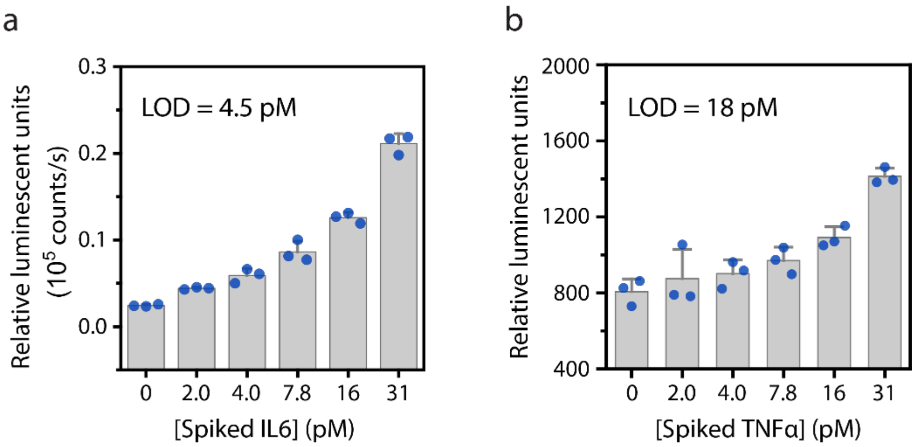

Figure S8. Limit-of-detection (LOD) measurement of the IL-6 and TNFα dRAPPID. (a) LOD determination of IL-6 dRAPPID. 0.1 nM Ab-LB and 1 nM Ab-SB were incubated (60 minutes) with different concentrations of IL-6. Before measuring the luminescent signal on a plate reader, 1000-fold diluted NLuc substrate was added to the mixture. (b) LOD determination of TNFα dRAPPID. 0.1 nM Ab-LB and 1 nM Ab-SB were incubated with low picomolar concentrations of TNFα. After 60 minutes, the substrate of NLuc (1000-fold final dilution) was added and the luminescent signal was measured with a plate reader. Measurements were done in buffer (PBS (pH 7.4), 0.1% (w/v) BSA). Data points represent technical replicates, with n = 3 independent preparations of target analyte.

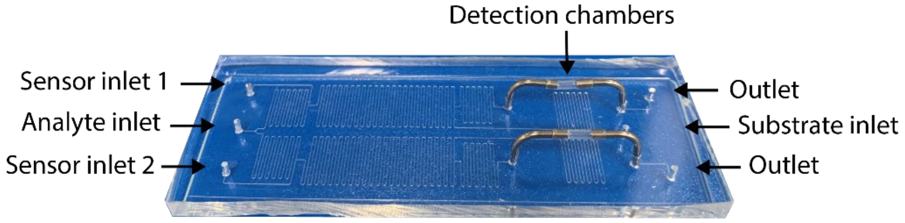

Figure S9. Picture of the PDMS dRAPPID microfluidic chip, with the two detection chambers, consisting of polyethylene tubing and two metal adapters. The blue or green luminescent signal, corresponding to analyte concentration, is captured with a digital camera in the two detection chambers. The chip has two sensor inlets, for two different dRAPPID assays, one analyte inlet, one substrate inlet (for the NLuc substrate) and two outlets.

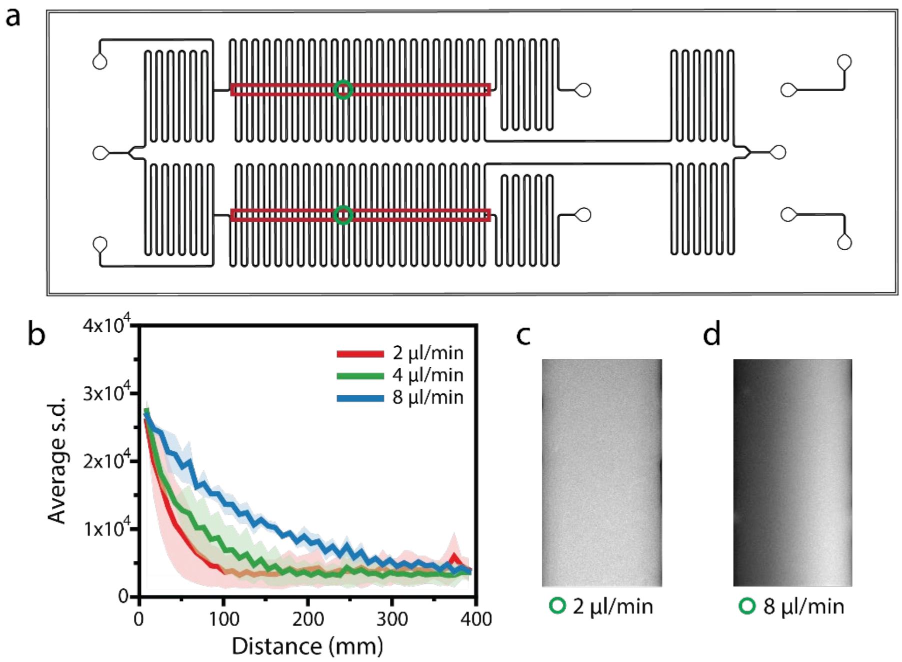

Figure S10. Homogenization of fluorescent signal in the serpentine channels. (a) Locations on the microfluidic chip where the fluorescent signal was measured. Data in (b) represents average s.d. measured in the parts of the channel in red boxes (in the middle of each turn in the serpentine channel). Data in (c) and (d) were measured in the green circle (the middle of the 20th turn in the serpentine channel). (b) Effect of flow rates on the mixing efficiency. 1.5 µM of calibrator luciferase was injected in the analyte inlet and buffer (PBS (pH 7.4), 0.1% (w/v) BSA) was added to the sensor inlet and substrate inlet. Red line represents flow rates of 1/2/4 µl/min, green line 2/4/8 µl/min and blue line 4/8/16 µl/min for sensor, analyte and substrate inlet, respectively, resulting in a flowrate in the serpentine mixing channel of 2 µl/min, 4 µl/min and 8 µl/min. Distance on the x-axis represents distance travelled from the analyte-sensor T-junction. The average standard deviation (y-axis) is derived from pictures taken in the red box in (a), in the middle of every turn in the serpentine channel. (c) Pictures of the serpentine channel with flow rates of 1/2/4 µl/min for sensor, analyte and substrate inlet, respectively. The measured location is depicted in (a) with the green circle. (d) Pictures of the serpentine channel with flow rates of 4/8/16 µl/min for sensor, analyte and substrate inlet, respectively. The measured location is depicted in (a) with the green circle. All pictures were taken with a Zeiss fluorescent microscope with an exposure time of 1.27 sec, a voltage time of 1.5 V, 200x magnification and a GFP filter.

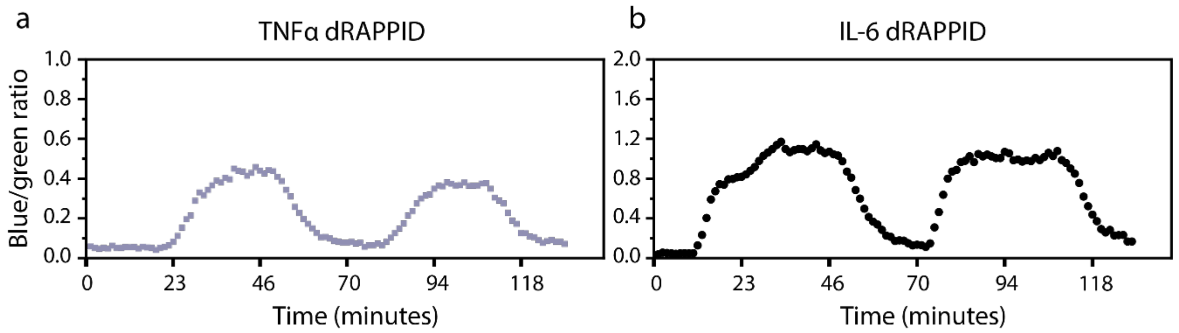

Figure S11. Normalized signal of continuous monitoring chip. 1 nM of Ab-LB and 10 nM of Ab-SB were injected in the lower sensor inlets and the green calibrator luciferase was added in the upper sensor inlet. (a) Normalized signal of the TNF*α* RAPPID from Figure 5a. The blue light of the RAPPID (upper detection chamber) was divided by the green light of the calibrator (lower detection chamber), generating a more stable signal over time. (b) Normalized signal of the IL-6 RAPPID from Figure 5b. The analyte-responsive blue light in the upper detection chamber was divided by the analyte-independent green light in the lower detection chamber, providing a stable signal over time.

References:

(1) Ni, Y.; Rosier, B. J. H. M.; van Aalen, E. A.; Hanckmann, E. T. L.; Biewenga, L.; Pistikou, A. M. M.; Timmermans, B.; Vu, C.; Roos, S.; Arts, R.; Li, W.; de Greef, T. F. A.; van Borren, M. M. G. J.; van Kuppeveld, F. J. M.; Bosch, B. J.; Merkx, M. A Plug-and-Play Platform of Ratiometric Bioluminescent Sensors for Homogeneous Immunoassays. *Nat Commun* **2021**, *12*, 4586.

(2) Geertjens, N. H. J.; de Vink, P. J.; Wezeman, T.; Markvoort, A. J.; Brunsveld, L. Straightforward Model Construction and Analysis of Multicomponent Biomolecular Systems in Equilibrium. RSC Chem. Biol. **2023**, 4, 252-260.

(3) Dixon, A. S.; Schwinn, M. K.; Hall, M. P.; Zimmerman, K.; Otto, P.; Lubben, T. H.; Butler, B. L.; Binkowski, B. F.; MacHleidt, T.; Kirkland, T. A.; Wood, M. G.; Eggers, C. T.; Encell, L. P.; Wood, K. v. NanoLuc Complementation Reporter Optimized for Accurate Measurement of Protein Interactions in Cells. *ACS Chem Biol* **2016**, *11* (2), 400–408.

(4) Ceballos-Alcantarilla, E.; Merkx, M. Understanding and Applications of Ser/Gly Linkers in Protein Engineering. In *Methods in Enzymology*; Merkx, M., Ed.; Academic Press Inc., **2021**; Vol. 647, pp 1–22.

(5) Kjaergaard, M.; Glavina, J.; Chemes, L. B. Predicting the Effect of Disordered Linkers on Effective Concentrations and Avidity with the "Ceff Calculator" App. In *Methods in Enzymology*; Merkx, M., Ed.; Academic Press Inc., **2021**; Vol. 647, pp 145–171.
